# Lnc-PFAR facilitates autophagy and exacerbates pancreatic fibrosis by reducing pre-miR-141 maturation in chronic pancreatitis

**DOI:** 10.1038/s41419-021-04236-z

**Published:** 2021-10-25

**Authors:** Tao Zhang, Guangquan Zhang, Wenbo Yang, Hongze Chen, Jisheng Hu, Zhongjie Zhao, Chundong Cheng, Guanqun Li, Yu Xie, Yilong Li, Rui Kong, Yongwei Wang, Gang Wang, Hua Chen, Xue-Wei Bai, Shangha Pan, Bei Sun, Le Li

**Affiliations:** 1 Department of Pancreatic and Biliary Surgery, The First Affiliated Hospital of Harbin Medical University, Harbin, Heilongjiang China; 2 Key Laboratory of Hepatosplenic Surgery, Ministry of Education, Harbin, Heilongjiang China

**Keywords:** RNAi, Diagnostic markers, Chronic pancreatitis

## Abstract

Chronic pancreatitis (CP) is described as progressive inflammatory fibrosis of pancreas, accompanied with irreversible impaired endocrine and exocrine insufficiency. Pancreatic stellate cells (PSCs) are widely distributed in the stroma of the pancreas and PSCs activation has been shown as one of the leading causes for pancreatic fibrosis. Our previous study has revealed that autophagy is dramatically activated in CP tissues, which facilitates PSCs activation and pancreatic fibrosis. Long non-coding RNAs (LncRNAs) have been recognized as crucial regulators for fibrosis-related diseases. LncRNAs interact with RNA binding protein or construct competitive endogenous RNA (ceRNA) hypothesis which elicited the fibrotic processes. Until now, the effects of lncRNAs on PSCs activation and pancreatic fibrosis have not been clearly explored. In this study, a novel lncRNA named Lnc-PFAR was found highly expressed in mouse and human CP tissues. Our data revealed that Lnc-PFAR facilitates PSCs activation and pancreatic fibrosis via RB1CC1-induced autophagy. Lnc-PFAR reduces miR-141 expression by suppressing pre-miR-141 maturation, which eventually upregulates the RB1CC1 and fibrosis-related indicators expression. Meanwhile, Lnc-PFAR enhanced PSCs activation and pancreatic fibrosis through trigging autophagy. Our study interrogates a novel lncRNA-induced mechanism in promoting the development of pancreatic fibrosis, and Lnc-PFAR is suggested to be a prospective therapeutic target in clinical scenarios.

## Introduction

Chronic pancreatitis (CP) is recognized as a pancreatic inflammatory disease, which is induced by genetic, environmental, alcohol, and other factors. Its pathological features include pancreatic acinar atrophy, destruction, interstitial fibrosis and eventually lead to endocrine and exocrine insufficiency of the pancreas [1–4]. The incidence of CP is 9.62/100,000 and the mortality rate is 9/10,000,000 [5]. Currently, no effective treatment has been shown to halt the pancreatic fibrotic process [6]. The pancreatic stellate cells (PSCs) are suggested as a critical component in the exacerbation of pancreatic fibrosis [7]. In the normal pancreas, PSCs are quietly localized in the area surrounding the acinus, showing droplets containing retinol and a small amount of extracellular matrix (ECM) proteins [8]. PSCs could be activated by inflammation, alcohol, trauma and are transformed into a myofibroblast-like phenotype, which are recognized by highly expressed alpha-smooth muscle actin (α-SMA), the presence of various growth factors (epidermal growth factor, vascular endothelial growth factor), cytokines (IL-6, IL-8, TGF-β) and a large number of ECM proteins [9]. Increased production of the ECM proteins fibronectin, periostin, matrix metalloproteinases (MMPs), and tissue inhibitors of matrix metalloproteinases (TIMPs) are the most common features exhibited by the activated PSCs phenotype. Hence, PSCs were described as the effector cells contributing to the stroma associated with CP [10]. The activated PSCs exhibit higher proliferation and migration capabilities, and participate in pancreatic fibrosis through improving energetic metabolism, cell death, and oxidative stress [11].

Autophagy is a conservative catabolic process that is involved in many physiological processes [12, 13]. During autophagy, cytoplasmic constituents are dissolved by lysosomal proteases, releasing amino acids, fatty acids, and glucose, which are the main metabolic substrates. In addition, autophagy helps to remove damaged organelles and cytoplasmic aggregates, thereby alleviate cell stress and maintain homeostasis [14, 15]. Several studies have demonstrated that autophagy could facilitate PSCs activation and pancreatic fibrogenesis. Sho Edno et al. reported that autophagy is required for PSCs activation, which promotes pancreatic cancer growth and metastasis by tumor-stromal interactions [8]. In addition, Li et al. revealed that hypoxia reduces stromal lumican and promotes tumor progression in pancreatic adenocarcinoma through autophagy-mediated degradation and reduction in protein synthesis within activated PSCs [16]. However, the mechanism of autophagy promoting pancreatic fibrosis by regulating PSCs activation still needs to be explored in depth.

Long non-coding RNAs (LncRNAs) are classified non-coding RNA as transcripts greater than 200 nt in length. LncRNAs have been shown to exert transcriptional, post-transcriptional, and epigenetic regulation of proteins, and could be used as a specific biomarker and therapeutic target for multiple diseases [17]. LncRNAs are involved in a variety of biological and pathological processes, including cell proliferation, apoptosis, survival, and differentiation [18, 19]. Emerging evidence has demonstrated that lncRNAs are engaging in the fibrosis of the liver, heart, and lung, and have been suggested to induce pancreatitis and pancreatic tumor progression [20–22]. Our previous study has indicated that low Linc-pint expression could be used as a biomarker for early diagnosing pancreatic cancer and predicting the prognosis [23]. In addition, the STX12 LncRNA axis has been shown to facilitate PSCs activation through subtype-specific sponge interaction with miR-148 [24]. Hence, the effects of lncRNAs on CP progression need to be further investigated.

In this study, transcriptome sequencing was performed to explore the differential lncRNAs between quiescent and activated PSCs. A pancreatic fibrosis-associated lncRNA, named Lnc-PFAR, was found to accelerate PSCs activation and pancreatic fibrosis in vivo and in vitro. Lnc-PFAR inhibited miR-141-5P expression level via binding to precursor miR-141 (pre-miR-141) and restrained its maturation. The downregulated miR-141-5P ultimately increased RB1CC1 expression and activated autophagy. Our study highlights the hallmark of Lnc-PFAR in PSCs activation and pancreatic fibrosis. It may provide a prospective biomarker for screening CP progression and a novel therapeutic target for the treatment.

## Results

### Lnc-PFAR is verified as a novel lncRNA in PSCs activation

**Fig. 1:**
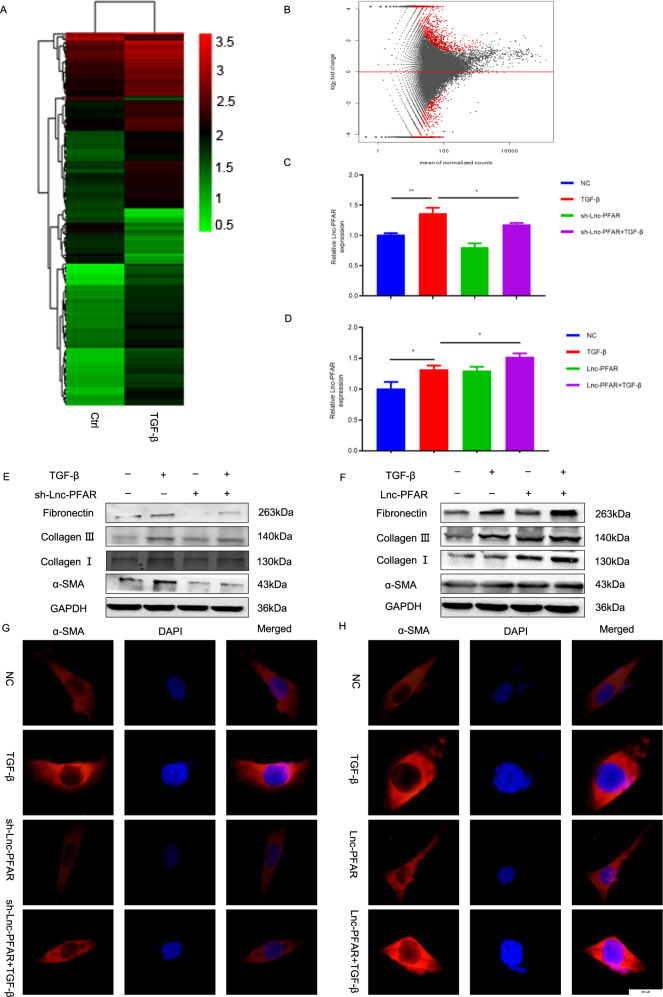
Lnc-PFAR was verified as a novel lncRNA in PSCs activation. **A**, **B** The heatmap and volcano plots showed that 189 genes were changed in activated PSCs compared with quiescent PSCs (fold change ≥ 2 or ≤ 0.5, *p* ≤ 0.05). **C**, **D** The expressions of Lnc-PFAR were compared in TGF-β-treated PSCs in combination with Lnc-PFAR downregulation or upregulation. **E**, **F** The expression levels of α-SMA, Collagen I, Collagen III, and Fibronectin were tested in PSCs in which Lnc-PFAR is downregulated or upregulated. The relative expression represents the ratio of target to GAPDH. **G**, **H** The α-SMA expressions were explored in PSCs of quiescent, TGF-β-treated, sh-Lnc-PFAR, sh-Lnc-PFAR plus TGF-β, Lnc-PFAR-upregulated, and Lnc-PFAR plus TGF-β groups via immunofluorescence staining (bars, 500 μm). The results are representative of three independent experiments (*p < 0.05 and **p < 0.01).

The activated PSCs play crucial roles in CP initiation and determine the severity of pancreatic fibrosis. The microarray analysis was used to investigate the differentially expressed lncRNAs in quiescent and activated PSCs in our study and a total of 189 lncRNAs (155 upwards, 34 downwards) were identified. Among them, pancreatic fibrosis-associated lncRNA (NONMMUT096607.1, start site 3217254 and end site 3218983) named Lnc-PFAR, was particularly prominent in PSCs activation. Lnc-PFAR has high homology in humans and mouse, indicating that it may contribute to human pancreatic fibrosis (Fig. 1A, B). Transforming growth factor-β (TGF-β) has been characterized as a key mediator for PSCs activation. To explore the effects of Lnc-PFAR in pancreatic fibrosis, the Lnc-PFAR expression was knocked down in PSCs by short hairpin RNA (shRNA) (Fig. S1A). The Lnc-PFAR expression level was decreased when Lnc-PFAR was inhibited. The opposite results were obtained in the upregulated studies (Fig. 1C, D). Meanwhile, the expressions of α-SMA, Collagen I, Collagen III, and Fibronectin were suppressed in Lnc-PFAR-silenced PSCs, while increased in the Lnc-PFAR overexpressed group (Fig. 1E, F, Fig. S1B, C). However, inhibition of Lnc-PFAR could not completely abrogate TGF-β-induced PSCs activation. These data indicated that Lnc-PFAR could not activate PSCs, but enhance TGF-β-mediated PSCs activation. Furthermore, the α-SMA expression was screened in different groups via IF staining. We found that knockdown of Lnc-PFAR restrained α-SMA expression (Fig. 1G, H, Fig. S1D, E). Similar results were confirmed in primary PSCs (Fig. S2A–F). Taken together, these results suggested that Lnc-PFAR induces pancreatic fibrosis via activating PSCs.

### Lnc-PFAR mediates miR-141-5p to activate PSCs

**Fig. 2:**
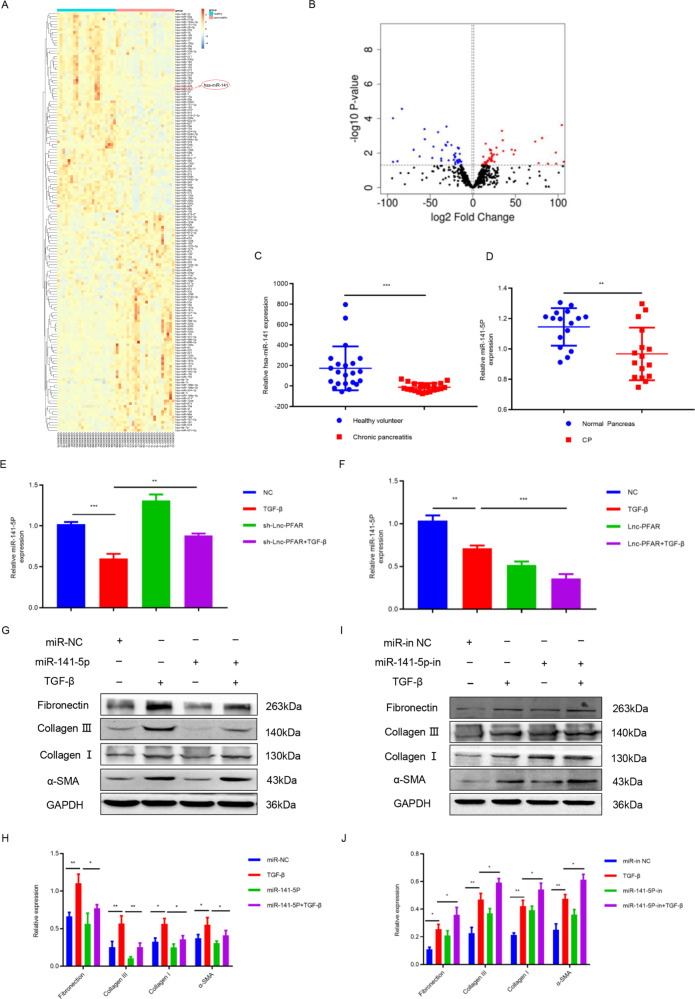
Lnc-PFAR inhibited miR-141-5p to promote PSCs activation. **A**, **B** The differentially expressed miRNAs in normal pancreas and CP tissues were identified from the GEO dataset (GSE24279). **C** The relative expression levels of miR-141 in normal pancreas and CP tissues in GSE24279. **D** The expression levels of miR-141 in 16 pairs of normal pancreas and CP tissues. **E**, **F** The expression levels of miR-141-5P were explored in PSCs of quiescent, TGF-β-treated, sh-Lnc-PFAR, sh-lnc-PFAR plus TGF-β, Lnc-PFAR, and Lnc-PFAR plus TGF-β groups. **G**–**J** The expressions of α-SMA, Collagen I, Collagen III, and Fibronectin were examined in PSCs of quiescent, TGF-β-treated, miR-141-5P mimic-treated, miR-141-5P mimic-treated plus TGF-β groups, miR-141-5P inhibitor-treated and miR-141-5P inhibitor-treated plus TGF-β groups. The relative expression represents the ratio of target to GAPDH. The results are representative of three independent experiments (*p < 0.05 and **p < 0.01).

MicroRNAs are recognized as significant indicators in fibrosis progression. Previous studies have demonstrated that lncRNAs participate in fibrotic processes in CP via the ceRNA mechanism [25]. To explore the effect of Lnc-PFAR in PSCs activation, differential miRNAs in paralleled human CP (*n* = 22) and normal pancreas (*n* = 22) tissues from the GEO dataset (GSE24279) were analyzed. A total of 157 different miRNAs, including 82 upregulated and 75 downregulated ones, were identified (Fig. 2A, B). As a member of the miR-200 family, miR-141 has been reported to engage in the fibrotic diseases development by autophagic activation [26, 27]. We then compared the levels of miR-141 in normal pancreatic tissues and CP tissues in the dataset, our results showed that miR-141 is dramatically downregulated in CP patients (Fig. 2C, D). To evaluate the regulatory role of Lnc-PFAR on miR-141 expression, the Lnc-PFAR levels were regulated, and the expressions of miR-141 were detected (Fig. 2E, F). Our results revealed that the miR-141-5p level was significantly decreased when Lnc-PFAR was upregulated. We hypothesized that Lnc-PFAR may promote PSCs activation via inhibiting miR-141-5p expression. Subsequently, the mimic or inhibitor of miR-141-5p were transfected and the expressions of α-SMA, Collagen I, Collagen III, and Fibronectin were tested (Fig. S3A, B). We found that downregulation of miR-141-5p promoted PSCs activation, while upregulation of miR-141 suppressed PSCs activation (Fig. 2G–J). The above data elucidated that Lnc-PFAR attenuates the inhibition of miR-141-5p, which may induce PSCs activation.

### Lnc-PFAR regulates miR-141-5p by stemming pre-miR-141 maturation

**Fig. 3:**
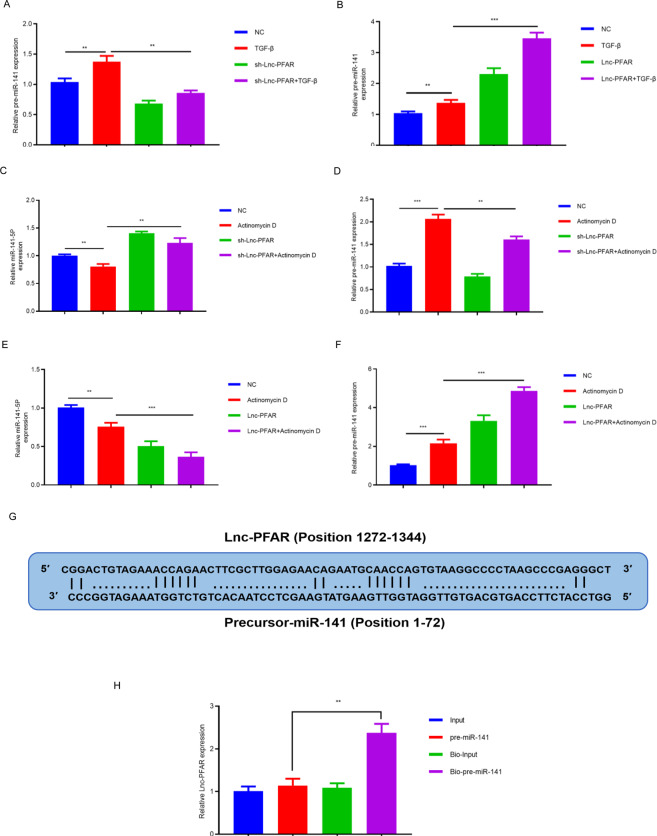
Lnc-PFAR restrained miR-141-5p by stemming pre-miR-141 maturation. **A**, **B** The expression levels of pre-miR-141 were explored in PSCs of quiescent, TGF-β-treated, sh-Lnc-PFAR, sh-Lnc-PFAR plus TGF-β, Lnc-PFAR, and Lnc-PFAR plus TGF-β groups. **C**–**F** The expression levels of pre-miR-141 were detected in PSCs of quiescent, actinomycin D-treated, sh-Lnc-PFAR, sh-Lnc-PFAR plus actinomycin D, Lnc-PFAR, and Lnc-PFAR plus actinomycin D groups. **G** Lnc-PFAR binds with pre-miR-141 and inhibits its maturation. **H** The binding sites between Lnc-PFAR and pre-miR-141 were identified by RNA pull down assay.

The conventional mechanism of lncRNAs restrains the expressions of miRNAs via the ceRNA hypothesis [28]. In addition, lncRNAs have been described to inhibit miRNA maturation by targeting pre-miRNAs or primary miRNAs (pri-miRNAs). Yu et al. have reported that CCAT2 selectively blocks the miR-145 maturation process, which decreases colon cancer proliferation and differentiation [28]. Xiao et al. have reported that lncRNA uc.173 promotes the renewal of the intestinal mucosa by destabilizing the pri-miR-195 transcript [29]. To further establish the effect of Lnc-PFAR on miR-141-5p maturation, the levels of pre-miR-141 and miR-141-5p were measured in PSCs when Lnc-PFAR levels were altered. The level of pre-miR-141 was significantly improved when Lnc-PFAR was upregulated. However, the expression of miR-141-5p was found to be negatively correlated with pre-miR-141 (Fig. 3A, B). We speculated that Lnc-PFAR may block miR-141-5p expression through suppressing pre-miR-141 maturation. Furthermore, an RNAs biosynthesis inhibitor, Actinomycin D was used to suppress pre-miR-141 maturation and we found that inhibition of Lnc-PFAR halted the maturity of pre-miR-141 (Fig. 3C–F). The pre-miR-141 showed perfect complementarity with Lnc-PFAR at 72 nucleotides situated for the whole length of pre-miR-141 (Lnc-PFAR position 1272–1344, pre-miR-141 position 1–72) (Fig. 3G). To validate the possible binding sites, a pre-miRNA pull-down assay was performed and higher levels of Lnc-PFAR were pulled down by bio-pre-miR-141 compared with negative control (Fig. 3H). Thus, we conclude that Lnc-PFAR binds with pre-miR-141, which impedes the maturity of pre-miR-141. In general, Lnc-PFAR ameliorates the miR-141-5p maturation process, which leashes PSCs activation and pancreatic fibrosis.

### miR-141-5p extenuates autophagy and suppresses PSCs activation through binding to RB1CC1

**Fig. 4:**
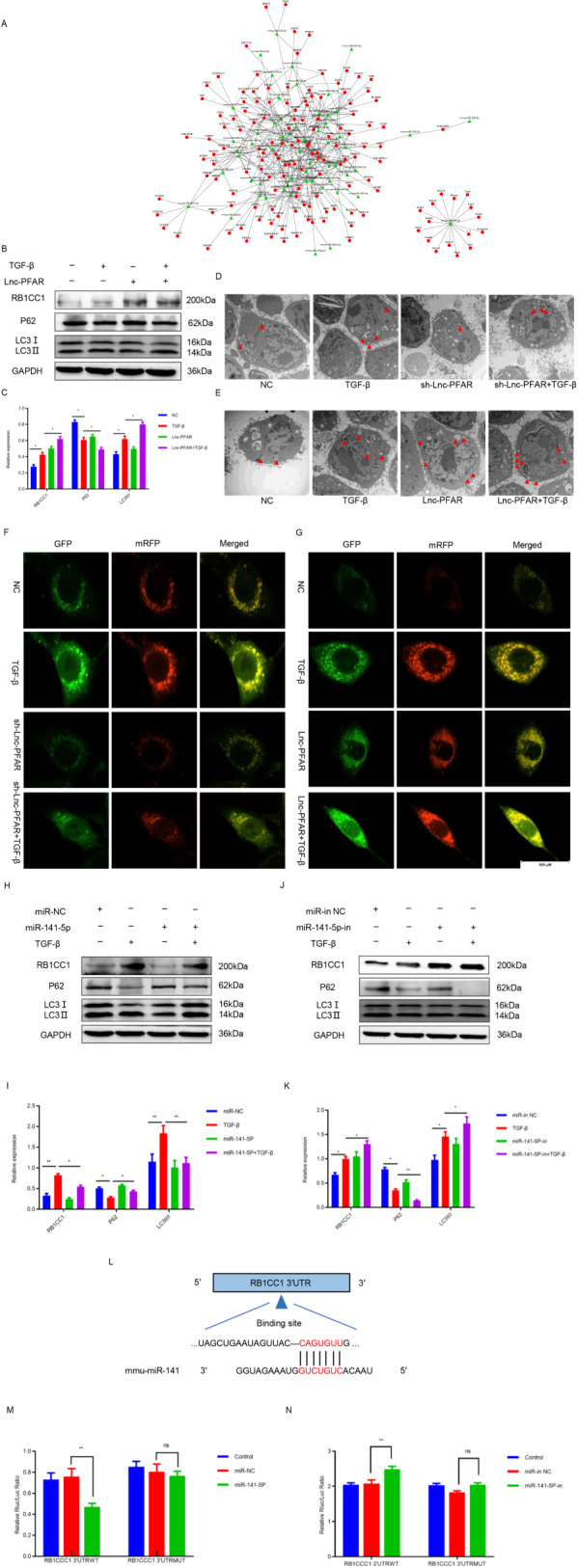
miR-141-5p extenuated autophagy and suppressed PSCs activation through binding to RB1CC1. **A** Construction of miRNA-mRNA regulatory network with differentially expressed miRNAs and mRNAs. **B**, **C** The expressions of RB1CC1, P62, and LC3II/I were tested in quiescent, TGF-β-treated, Lnc-PFAR, and Lnc-PFAR plus TGF-β groups. The relative expression represents the ratio of target to GAPDH. **D**, **E** The TEM assays showed the autophagic levels in quiescent and TGF-β-treated, sh-Lnc-PFAR, sh-Lnc-PFAR plus TGF-β Lnc-PFAR and Lnc-PFAR plus TGF-β-treated groups (bars, 2 μm). **F**, **G** The immunofluorescence assays were assessed to screen the autophagic flux in eight different groups (bars, 500 μm). **H**–**K** The expressions of RB1CC1, P62, and LC3II/I were detected in PSCs of quiescent, TGF-β-treated, miR-141-5P mimic-treated, miR-141-5P mimic-treated plus TGF-β groups, miR-141-5P inhibitor-treated and miR-141-5P inhibitor-treated plus TGF-β groups. The relative expression represents the ratio of target to GAPDH. **L** Schematic diagram of the luciferase reporter plasmids of wild type-RB1CC1 3′ UTR. **M**, **N** Luciferase activity assays were performed to confirm the interaction between miR-141-5p and RB1CC1. The results are representative of three independent experiments (*p < 0.05 and **p < 0.01).

It is well established that miRNAs induce mRNA degradation by binding to 3′ untranslated regions (3′UTR). Based on the identified 433 differential mRNAs between activated and quiescent PSCs, the miRNA-mRNA regulatory network was built. Our data indicated that miR-141-5p may interact with Retinoblastoma coiled-coil protein 1 (RB1CC1) and expedite PSCs activation (Fig. 4A). Our previous data have demonstrated that RB1CC1 induces ULK1 dephosphorylation and enhances autophagy, which promotes PSCs activation and pancreatic fibrosis [30]. Furthermore, the RB1CC1 expression and autophagic level were explored. The results showed that both were increased when Lnc-PFAR was upregulated (Fig. 4B, C). The transmission electron microscopy (TEM) and mRFP-GFP-LC3 assay were used to access the autophagosome and autophagic flux. We found that autophagy was activated when Lnc-PFAR was upregulated (Fig. 4D–G, Fig. S4A, B). Next, the level of RB1CC1, autophagic indicators and PSCs activation were explored when the miR-141-5p mimic or inhibitor were transfected. The results revealed that miR-141-5p mimic inhibited PSCs activation by attenuating RB1CC1 expression and autophagy, while inhibition of miR-141-5p enhanced PSCs activation via upregulating RB1CC1-induced autophagy (Fig. 4H–K). Furthermore, the luciferase assay was used to verify the binding site between miR-141-5p and RB1CC1 3′UTR (Fig. 4L). We found that the luciferase activity of the WT 3′-UTR RB1CC1 was significantly decreased when the miR-141-5p mimic was transfected (Fig. 4M, N). Above all, these results revealed that miR-141-5p inhibits autophagy and PSCs activation through binding to RB1CC1.

### Lnc-PFAR enhances pancreatic fibrosis in vivo

**Fig. 5:**
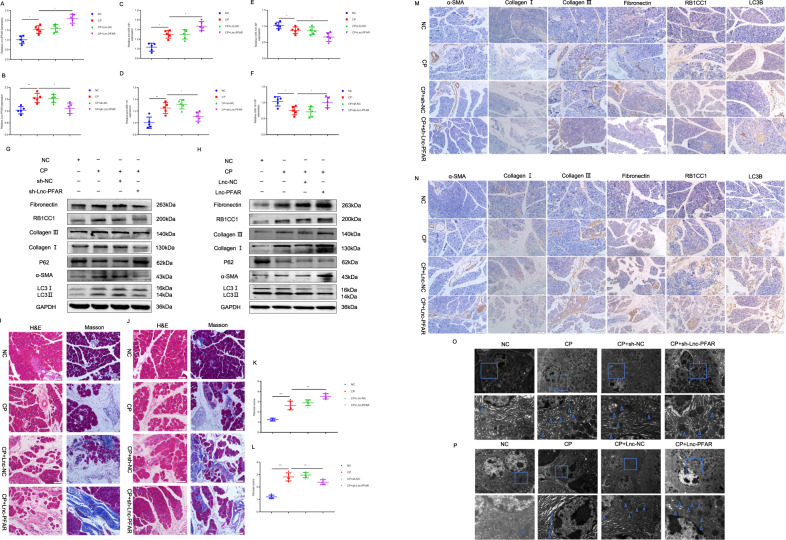
Lnc-PFAR enhanced pancreatic fibrosis in vivo. **A**–**F** The expression levels of Lnc-PFAR, pre-miR-141, and miR-141-5P were explored in pancreatic tissues of negative control, CP, CP plus sh-Lnc-PFAR injection and CP plus Lnc-PFAR injection groups. **G**–**H** The expressions of α-SMA, Collagen I, Collagen III, Fibronectin, RB1CC1, P62, and LC3II/I were tested in the negative control, CP, sh-Lnc-PFAR, CP plus sh-Lnc-PFAR, Lnc-PFAR, and CP plus Lnc-PFAR groups. The relative expression represents the ratio of target to GAPDH. **I**, **J** The H&E and Masson assays were conducted to indicate CP and pancreatic fibrosis in mouse models (*n* = 5) (original magnification, 20×, bars, 50 μm). **K**, **L** The different pancreatic fibrosis scores were compared among negative control, CP, sh-Lnc-PFAR, and CP plus sh-Lnc-PFAR, Lnc-PFAR, and CP plus Lnc-PFAR groups. **M**, **N** The expressions of α-SMA, Collagen I, Collagen III, Fibronectin, RB1CC1, P62, and LC3II/I were analyzed by immunohistochemistry (original magnification, 20×, bars, 50 μm) in different groups. **O**–**P** The TEM assays revealed the autophagic levels in different groups (bars, 2 μm).

The upregulated and downregulated Lnc-PFAR lentivirus were generated to investigate the role of Lnc-PFAR in pancreatic fibrosis in a mouse model (Fig. S5A). The expressions of Lnc-PFAR, miR-141-5p, and pre-miR-141 in pancreatic tissues were detected. Inhibition of Lnc-PFAR impaired the level of pre-miR-141 and improved miR-141-5p expression. The opposite results were confirmed in Lnc-PFAR-upregulated groups (Fig. 5A–F, Fig. S5B–G). We then explored the relative expressions among Lnc-PFAR, miR-141-5p, and pre-miR-141, and no correlation was found in tissues or plasma samples (Fig. S6A–D). Furthermore, suppression of Lnc-PFAR reduced the fibrotic indicators, including α-SMA, Collagen I, Collagen III, and Fibronectin (Fig. 5G, H, M, N, Fig. S7A, B). Our data revealed that inhibition of Lnc-PFAR alleviated pancreatic fibrosis and CP progression (Fig. 5I–L). The expressions of RB1CC1 and LC3B were impeded due to the inhibition of autophagy, and overexpression of Lnc-PFAR ameliorated those effects (Fig. 5G, H, M, N). Additionally, we found that the levels of Lnc-PFAR and pre-miR-141 were elevated in CP mice and intraperitoneal injection of sh-Lnc-PFAR lentivirus blocked pre-miR-141 expression and enhanced the miR-141-5p expression in pancreatic tissues. The expressions of α-SMA and other fibrosis-related indicators were screened via qRT-PCR assay. Lnc-PFAR improved α-SMA expression in CP tissues, but no significant correlation was found between α-SMA and Lnc-PFAR levels (Fig. S7C–F). Meanwhile, the autophagic level was examined in mouse models and the results suggested that Lnc-PFAR improved the formation of autophagosome (Fig. 5O–P). In addition, the same results were obtained in the caerulein-injection CP model (X6 injections, three times a week, three weeks) (Fig. S8A–D). Collectively, Lnc-PFAR exacerbates pancreatic fibrosis via accelerating autophagy and PSCs activation, Lnc-PFAR could be used as a therapeutic effect for relieving pancreatic fibrosis.

### Lnc-PFAR expression is correlated with pancreatic fibrosis in CP patients

**Fig. 6:**
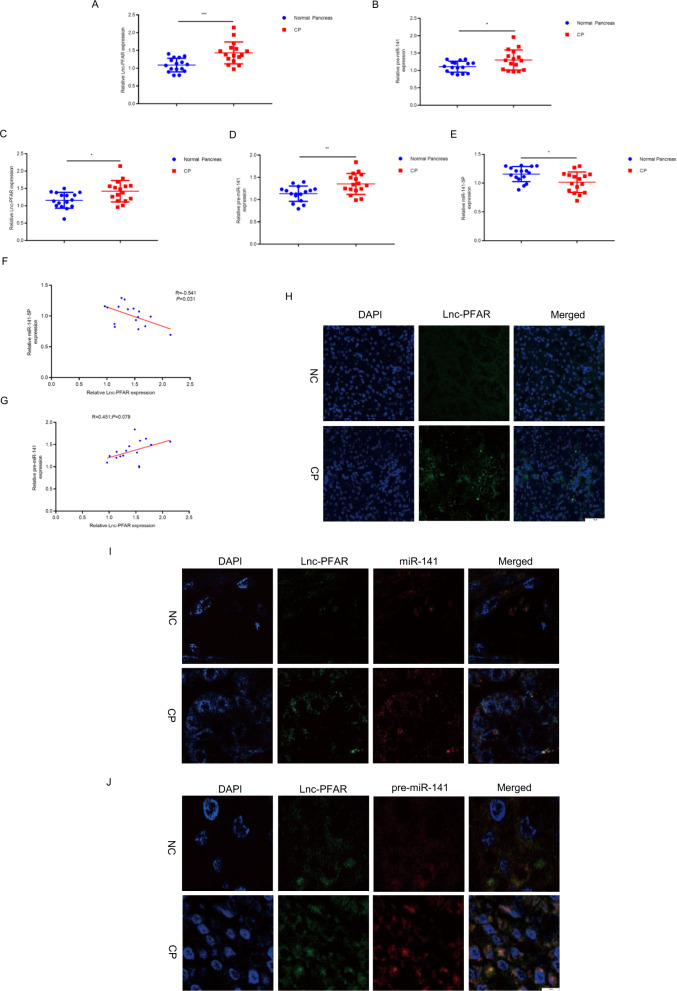
Lnc-PFAR expression was correlated with pancreatic fibrosis in CP patients. **A**, **B** The expression levels of lnc-PFAR and pre-miR-141 were explored in human normal pancreas and CP tissues. **C**–**E** The expression levels of lnc-PFAR, pre-miR-141, and miR-141-5P were measured in the plasma of healthy volunteers and CP patients. **F**, **G** The correlations among Lnc-PFAR, pre-miR-141, and miR-141-5P expression levels were explored in the plasma of healthy volunteers and CP patients. **H** FISH assay showed that Lnc-PFAR was predominantly localized in the cytoplasm. **I** FISH assay demonstrated that miR-141 and Lnc-PFAR were mutually exclusive in the cytoplasm in the normal pancreas and CP tissues. **J** FISH assay indicated the co-localization between Lnc-PFAR and pre-miR-141 in normal pancreas and CP tissues(bars, 50 μm).

We examined the levels of Lnc-PFAR and pre-miR-141 in normal and CP tissues (Fig. 6A, B). Our data showed that the expressions of Lnc-PFAR and pre-miR-141 were increased while the miR-141-5p level was decreased in CP tissues. There was no correlation among Lnc-PFAR, miR-141-5P, and pre-miR-141 in CP tissues (Fig. S9A, B). Similarly, the above indicators were tested in the plasma of healthy volunteers and CP patients, and higher levels of Lnc-PFAR, pre-miR-141, and lower miR-141-5P expression were found in CP patients (Fig. 6C–E). The slight significant correlations between Lnc-PFAR and miR-141-5P, Lnc-PFAR and pre-miR-141 in CP patients’ plasma were found (Fig. 6F, G). Furthermore, FISH assay was performed to explore the localizations and expressions of Lnc-PFAR, miR-141, and pre-miR-141 in normal and CP tissues. We found that Lnc-PFAR was predominantly located in the cytoplasm and highly expressed in CP tissues than the normal pancreas. Lnc-PFAR and pre-miR-141 were co-localized in the cytoplasm and Lnc-PFAR and miR-141-5P were mutually exclusive (Fig. 6H–J). CP has been shown to induce a higher incidence of pancreatic cancer (PC). We then compared the expression of Lnc-PFAR in CP and PC tissues. Our results indicated a lower Lnc-PFAR level in PC tissues than that in CP tissues (Figure S9C). Taken together, highly expression of Lnc-PFAR could be used for predicting the severity of pancreatic fibrosis and it could serve as an effective biomarker for the early detection of CP.

## Discussion

CP is a pancreatic fibro-inflammatory syndrome in individuals with genetic, environmental and/or other risk factors which develop persistent pathologic responses to parenchymal injury or stress [3]. The activation of PSCs is considered a core process in pancreatic fibrogenesis. In our study, we propose a new LncRNA, named Lnc-PFAR, which is differentially expressed between quiescent and activated PSCs. Lnc-PFAR locates at 15q with a length of 1729 nt and is highly expressed in activated PSCs. The upregulated Lnc-PFAR exacerbates pancreatic fibrosis through activating autophagy. Besides, the upregulation of Lnc-PFAR promotes activated PSCs proliferation and migration (Fig. S10A–F). Our study corroborates the hypothesis of Lnc-PFAR suppressing pre-miR-141 maturation which accelerates PSCs activation by triggering autophagy. To explore the colocalizations and expressions of Lnc-PFAR, miR-141, and pre-miR-141 between quiescent PSCs and activated PSCs groups, the FISH assay showed that Lnc-PFAR was predominantly located in the cytoplasm and highly expressed in activated PSCs. Additionally, Lnc-PFAR and pre-miR-141 were co-localized in the cytoplasm, and Lnc-PFAR and miR-141-5P were mutually exclusive in the cytoplasm (Fig. S11A, B). In our previous study, RB1CC1 has been demonstrated to enhance TGF-β-induced PSCs activation. Here, Lnc-PFAR was shown to inhibit miR-141-5P expression via binding with pre-miR-141 and to restrain its maturation. The downregulated miR-141-5P ultimately promotes the transcription of RB1CC1 in the downstream region. This study clarifies the vital role of Lnc-PFAR in pancreatic fibrosis and provides novel insights for discovering potential therapeutic targets for CP.

LncRNAs exert different types of mechanisms through the ceRNA regulatory network, interaction with RBPs, and genes inactivation or degradation (such as chromatin remodeling, DNA methylation, RNA decay, and histone protein modification) [28]. In recent years, the effects of lncRNA in stellate cells (including pancreatic stellate cells and hepatic stellate cells) have been gradually explored [24, 25, 31, 32]. Liu et al. demonstrated LncRNA MIT enhances PSCs activation through regulating the miR‐216a‐3p/COX‐2 axis [25]. Yu et al. revealed that Lnc-SNHG7 reduces miR-378a-3p and attenuates its control on DVL2, leading to aberrant Wnt/β-catenin activity, which contributes to HSCs activation and liver fibrosis progression [31]. The identification of the ceRNA hypothesis expands the theory of the dynamics and lncRNA-disease network and provides more strategies for PSCs activation and CP diagnosis. In this study, we propose a relatively novel mechanism that Lnc-PFAR exerts its biological effect of PSCs activation and pancreatic fibrosis expedition by suppressing pre-miR-141 maturation.

MiRNAs are small (21–23 nt) non-coding RNA molecules that regulate mRNA degradation by interacting with 3′UTR and driving the translational suppression [33]. MiR-141 is a member of the miR-200 family and has been reported to activate autophagy in fibrotic diseases and cancers [26, 27]. Li et al. reported that triptolide alleviates fibrosis by restoring autophagy through the miR-141-3p/PTEN/Akt/mTOR pathway [34]. Qian et al. indicated that miR-141-3P blockes TGF-β1 induced EMT and pulmonary fibrosis via targeting ZEB1 [26]. Che et al. found that melatonin abrogates cardiac fibrosis via inhibiting MALAT1/miR-141-mediated NLRP3 inflammasome activation and TGF-β1/Smads signaling [35]. However, the effects of miR-141 in multifactorial pancreatic fibrosis are not clear. In this study, Lnc-PFAR shows the capacity of enhancing PSCs activation through inhibiting miR-141-5p biogenesis. Despite of the inconsistent sequences between Lnc-PFAR and miR-141-5p, it is impossible to verify that Lnc-PFAR restrains the expression of miR-141-5p through the conventional ceRNA mechanism. Accumulating evidence shows the participation of lncRNAs in the splicing of miRNA precursors. Our study identifies the definitive binding sites in pre-miR-141 which have sufficient complementarity to Lnc-PFAR, and firstly demonstrates that Lnc-PFAR impairs pre-miR-141 maturation to exacerbate CP progression.

Autophagy is a cellular pathway involved in protein and organelle degradation, and plays an essential role in maintaining cellular homeostasis [12, 36–38]. There are roughly three classes of autophagy, including macroautophagy, microautophagy, and chaperone-mediated autophagy. Macroautophagy is a major type of autophagy, which has been extensively studied compared to microautophagy and chaperone-mediated autophagy [15]. Herein, we refer to macroautophagy simply as “autophagy”. Previous studies have revealed that autophagy participates in PSCs activation. Sho Endo et al. demonstrated that autophagy improves PSCs activation and promotes pancreatic cancer growth and metastasis through tumor-stromal interactions. They also found that genetic and chemical autophagic inhibitor maintain PSCs to be a quiescent state [8]. Yan et al. indicated that a combination of ERK inhibitor and autophagy inhibitor could suppress PSCs activation, cancer-stromal interaction, and metastasis [39]. Li et al. revealed that hypoxia promotes tumor progression in pancreatic adenocarcinoma through autophagy-mediated degradation and reduction of lumican within activated PSCs [16]. Li et al. confirmed that inhibition of autophagy suppresses PSCs activation and increases ECM degradation by decreasing the expression of TGF-β1 and altering the MMP/TIMP ratio [40]. Meanwhile, our previous study highlighted that upregulated RB1CC1 exacerbates pancreatic fibrosis through promoting autophagic activation. Currently, our study illustrates the possible molecular mechanism of lncRNA inducing PSCs activation and pancreatic fibrosis through autophagy, which brings potential value and clinical significance for the therapeutic of CP.

**Fig. 7:**
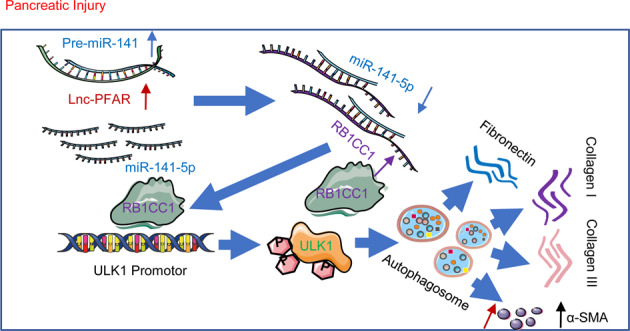
Schematic presentation of the mechanism of Lnc-PFAR promoting pancreatic fibrosis development. Lnc-PFAR decreased the expression of miR-141 by inhibiting pre-miR-141 maturation, the reduction of miR-141-5P released RB1CC1, and then increased ULK1 expression. Eventually, the ULK1-induced autophagy aggravated PSCs activation and pancreatic fibrosis.

In conclusion, this study provides new insights into the effects of Lnc-PFAR-induced autophagy through enhancing RB1CC1 expression in pancreatic fibrosis. Lnc-PFAR decreased the expression of miR-141 by inhibiting pre-miR-141 maturation, the reduction of miR-141-5P released RB1CC1, and then increased ULK1 expression. Eventually, the ULK1-induced autophagy aggravated PSCs activation and pancreatic fibrosis (Fig. 7). This study provides evidence for the involvement of Lnc-PFAR in CP and its valuable for future investigations and clinical instruction.

## Materials and methods

### Clinical samples and plasma

Tissues of 16 normal individuals, 16 CP patients, and 10 PC patients and plasma of 16 normal individuals and 16 CP patients were obtained from benign pancreatic cystic neoplasm (PCN) patients and CP patients in the Department of Pancreatic and Biliary Surgery, The First Affiliated Hospital of Harbin Medical University from January 2017 to January 2019. The information of all the patients are listed in Supplementary Table S1. The informed consent was obtained from patients and the study was approved by the review committee of the First Affiliated Hospital of Harbin Medical University.

### Reagents and chemicals

The TGF-β was purchased from Peperotech (Rocky Hill, USA). The cerulein, Actinomycin D were ordered from Sigma (Sigma-Aldrich, Shanghai, China). The pcDNA3.1-Lnc-PFAR was obtained from GenScript (Nanjing, China). The sh-Lnc-PFAR vector and lentiviruses of sh-Lnc-PFAR were purchased from Genechem (Shanghai, China).

### Cell culture

Mice PSCs lines were purchased from Cell Bank of the Chinese Academy of Science. PSCs were cultured in Dulbecco’s modified Eagle’s medium (Gibco, Gaithersburg, USA), supplemented with 10% fetal bovine serum (Gibco), 1% penicillin, and streptomycin at 37 °C with 5% CO_2_.

### Mice

Eight weeks old male C57BL/6 mice were purchased from the Experimental Animal Center of The Second Affiliated Hospital of Harbin Medical University. Mice were randomly divided into eight groups (negative control, CP, CP plus sh-negative control and CP plus sh-Lnc-PFAR, negative control, CP, CP plus Lnc-negative control, and CP plus Lnc-PFAR). CP was induced by ligating the pancreatic duct at the junction of the common duct and pancreatic duct. The bile duct and concomitant artery were spared. The eight groups of mice expect the negative control group received a single intraperitoneal injection of cerulein (50 mg/kg/body weight) 2 days after the pancreatic duct ligation [41]. In the CP plus sh-Lnc-PFAR and CP plus Lnc-PFAR groups, the mice also received a single intraperitoneal injection of a lentiviral vector of sh-Lnc-PFAR or Lnc-PFAR at the second day after ligation. All of the mice were killed at 21 days after pancreatic duct ligation [42, 43]. This study protocol was approved by the Institutional Review Board of The First Affiliated Hospital of Harbin Medical University.

### Hematoxylin and eosin staining (H&E), masson staining, and Immunohistochemistry (IHC)

The hematoxylin and eosin (H&E) staining and immunohistochemical staining protocols were described previously [44, 45]. Masson staining was performed following the manufacture’s protocol (Baso, Zhuhai, China). In the immunohistochemical staining, paraffin-embedded tissue sections (5 μm) were stained with anti-Fibronectin, anti-RB1CC1, anti-Collagen I, anti-Collagen III, anti-α-SMA, and anti-LC3B, The number of positive cells was counted in five randomly selected microscopic fields (×20, Nikon, Japan).

### mRFP-GFP-LC3 assay

The mRFP-GFP-LC3 assay was performed as described previously [46, 47]. PSCs were transfected with GFP-mRFP-LC3 lentiviral vector and selected by puromycin. The stably transfected cells were transfected with sh-Lnc-PFAR or Lnc-PFAR vector and were viewed under a fluorescence microscope. The number of GFP and mRFP dots was determined by manual counting of the fluorescent puncta in five high-power fields (original magnification, ×40, Olympus, Japan).

### Transmission electron microscope

Transmission electron microscopy was performed as described previously [48]. The PSCs transfected with sh-Lnc-PFAR and Lnc-PFAR were fixed in 2.5% glutaraldehyde. Fresh tissues were also fixed in 2.5% glutaraldehyde, and post-fixed in 1% osmium tetroxide buffer. Cells and tissues were embedded in spur resin and thin sections were cut. The sectioned grids were stained with a saturated solution of uranyl acetate and lead citrate. Sections were examined at 80 kV using a JEOL 1200EX transmission electron microscope (Harbin Medical University, China).

### Luciferase reporter assay

To detect the interaction between RB1CC1 and miR-141-5p, the full-length 1847 bp 3′UTR of wild-RB1CC1 (WT) and same length mutant-RB1CC1 (MUT) were amplified and then cloned into pmiR-Report Luciferase vector (GenScript, Nanjing, China). The PSCs were co-transfected 50 nM miR-141-5P mimic or 100 nM miR-141-5p inhibitor with 500 ng of Luciferase constructs according to the manufacturer’s protocol. The cells were harvested 24 h after transfection, and the luciferase activity was measured with a Dual-Luciferase Reporter Assay System (Promega, Madison, USA), through Varioskan Flash Spectral Scanning Multimode Reader (Thermo Fisher Scientific, Massachusetts, USA). Firefly luciferase activity was used to normalize the transfection efficiency. The sequences were listed in Supplementary Table S2.

### RNA isolation, reverse-transcription and quantitative realtime polymerase Chain Reaction (qRT-PCR)

RNA isolation and the PCR amplification conditions were followed as previously described [23]. The qRT-PCR assay (SRBY Green) was performed on Applied Biosystem 7500. The relative expression levels of LncRNA, mRNAs, and miRNA were calculated and quantified using the 2^−ΔΔCT^ method. GAPDH and U6 served as the endogenous control respectively. The primer sequences were designed by Primer 5.0 and are listed in Supplementary Table S3.

### Immunofluorescence

Immunofluorescence was performed as described previously [49]. In brief, activated PSCs transfected with sh-Lnc-PFAR and Lnc-PFAR plasmid were seeded on 24-well plates. The cells were fixed with 4% paraformaldehyde for 30 min and were permeabilized with 0.5% Triton X-100 for 20 min. After incubation for 2 h with anti-α-SMA (Cell Signaling Technology), the cells were washed with PBS for three times. Then, the cells were incubated with secondary antibodies for 1 h (Beyotime, Nanjing, China), and 4′6-diamino-2-phenylindole (DAPI, Beyotime) was added to stain the cell nuclei. The cells were detected by a laser scanning confocal microscope (×40, Olympus, Japan).

### RNA pull down assay

Biotin-labeled pre-miR-141 and control pre-miR-141 were transfected and whole-cell lysates were collected 48 h later. The lysates were then mixed with Streptavidin-Dynal beads and incubated for overnight [29]. The beads-bound RNA was isolated and analyzed by the qRT-PCR assay. Input RNA was extracted and served as a negative control.

### Fluorescence in situ hybridization (FISH)

The paraffin sections were baked in an oven at 60 °C for 1 h, then dewaxed in xylene, and then proteinase K digestion for 10 min at 55 °C. The sections were placed in different pretreatment solutions in sequence, and the sections were finally washed with deionized water. The hybridization mixture was dropped on the sections, incubated at 37 °C for 2 h, and the buffer was washed and placed in different ampicillin [50]. Counter-staining with hematoxylin and photos were taken with a laser confocal microscope (×40, Olympus, Japan).

### Western blot analysis

Western blot analysis was performed as described previously [46, 51]. Whole-cell lysates with approximately 40 μg of proteins were resolved on 10 and 12% SDS-PAGE and were subjected to western blot assay using the antibodies listed in Supplementary Materials. After appropriate secondary antibody incubation, the bands were visualized with the Molecular Imager System (BIO-RAD, Hercules, USA) using an enhanced chemiluminescence method (Thermo Fisher Scientific, Massachusetts, USA). The antibodies are listed in Supplementary Table S4.

### Statistical analysis

Results are shown as the mean ± SD. Statistical analysis was performed with Graphpad 7 software and analysis of variance (ANOVA) and a Student’s t-test were used to evaluate statistical significance. The correlations were analyzed using Pearson’s correlation coefficients. Differences are considered significant when *p < 0.05, **p < 0.01, ***p < 0.001 and ns *p* > 0.05.

## Supplementary information

supplemental information

Figure S1

Figure S2

Figure S3

Figure S4

Figure S5

Figure S6

Figure S7

Figure S8

Figure S9

Figure S10

Figure S11

## Data Availability

Data required to support the findings of this study are present in the main text or supplementary materials. All other data supporting the findings of this study are available from the corresponding authors upon reasonable request.
