# Supplementary material for: Lnc-PFAR facilitates autophagy and exacerbates pancreatic fibrosis by reducing pre-miR-141 maturation in chronic pancreatitis
Source: Cell Death Dis. 2021 Oct 25;12(11):996. doi: 10.1038/s41419-021-04236-z (PMC8547218; doi:10.1038/s41419-021-04236-z)

Table S1. Characteristics of pancreas donors of normal controls, CP and PC patients**.**

| **Donors** | **Age（years）** | **Sex** | **Pathological Diagnosis** |
| --- | --- | --- | --- |
| **Normal control 1** | **49** | **Male** | **pancreatic cystic neoplasm** |
| **Normal control 2** | **50** | **Male** | **pancreatic cystic neoplasm** |
| **Normal control 3** | **44** | **Female** | **pancreatic cystic neoplasm** |
| **Normal control 4** | **42** | **Male** | **pancreatic cystic neoplasm** |
| **Normal control 5** | **44** | **Male** | **pancreatic cystic neoplasm** |
| **Normal control 6** | **50** | **Male** | **pancreatic cystic neoplasm** |
| **Normal control 7** | **36** | **Female** | **pancreatic cystic neoplasm** |
| **Normal control 8** | **45** | **Male** | **pancreatic cystic neoplasm** |
| **Normal control 9** | **42** | **Male** | **pancreatic cystic neoplasm** |
| **Normal control 10** | **38** | **Male** | **pancreatic cystic neoplasm** |
| **Normal control 11** | **40** | **Female** | **pancreatic cystic neoplasm** |
| **Normal control 12** | **45** | **Female** | **pancreatic cystic neoplasm** |
| **Normal control 13** | **56** | **Male** | **pancreatic cystic neoplasm** |
| **Normal control 14** | **48** | **Male** | **pancreatic cystic neoplasm** |
| **Normal control 15** | **45** | **Male** | **pancreatic cystic neoplasm** |
| **Normal control 16** | **38** | **Female** | **pancreatic cystic neoplasm** |
| **CP patient 1** | **47** | **Male** | **Chronic pancreatitis** |
| **CP patient 2** | **49** | **Male** | **Chronic pancreatitis** |
| **CP patient 3** | **66** | **Male** | **Chronic pancreatitis** |
| **CP patient 4** | **62** | **Male** | **Chronic pancreatitis** |
| **CP patient 5** | **32** | **Male** | **Chronic pancreatitis** |
| **CP patient 6** | **55** | **Male** | **Chronic pancreatitis** |
| **CP patient 7** | **50** | **Male** | **Chronic pancreatitis** |
| **CP patient 8** | **59** | **Male** | **Chronic pancreatitis** |
| **CP patient 9** | **62** | **Male** | **Chronic pancreatitis** |
| **CP patient 10** | **55** | **Female** | **Chronic pancreatitis** |
| **CP patient 11** | **42** | **Male** | **Chronic pancreatitis** |
| **CP patient 12** | **54** | **Male** | **Chronic pancreatitis** |
| **CP patient 13** | **46** | **Female** | **Chronic pancreatitis** |
| **CP patient 14** | **51** | **Male** | **Chronic pancreatitis** |
| **CP patient 15** | **39** | **Female** | **Chronic pancreatitis** |
| **CP patient 16** | **49** | **Male** | **Chronic pancreatitis** |
| **PC patient 1** | **56** | **Male** | **Pancreatic cancer** |
| **PC patient 2** | **62** | **Male** | **Pancreatic cancer** |
| **PC patient 3** | **58** | **Female** | **Pancreatic cancer** |
| **PC patient 4** | **64** | **Male** | **Pancreatic cancer** |
| **PC patient 5** | **49** | **Female** | **Pancreatic cancer** |
| **PC patient 6** | **58** | **Female** | **Pancreatic cancer** |
| **PC patient 7** | **62** | **Male** | **Pancreatic cancer** |
| **PC patient 8** | **66** | **Female** | **Pancreatic cancer** |
| **PC patient 9** | **71** | **Male** | **Pancreatic cancer** |
| **PC patient 10** | **65** | **Male** | **Pancreatic cancer** |

**Table S2. Sequences of WT-RB1CC1, MUT-RB1CC1 and miR-141**

| Name: | Sequences (5’ to 3’) |
| --- | --- |
| WT-RB1CC1 | UAGCUGAAUAGUUAC---CAGUGUUG |
| MUT-RB1CC1 | UAGCUGAAUAGUUAC---GUCUGUCG |
| mmu-miR-141 | GGUAGAAAUGGUCUGUCACAAU(3’to5’) |

**Table S3. Sequences of primers used in RT-PCR**

| Primer Name: | Sequences (5’ to 3’) |
| --- | --- |
| Mus-GAPDH-Forward | **CCTCTGACTTCAACAGCGACCAC** |
| Mus-GAPDH-Reverse  Human-GAPDH-Forward  Human-GAPDH-Reverse | **TGGTCCAGGGGTCTTACTCC**  **GTCTCCTCTGACTTCAACAGCG**  **ACCACCCTGTTGCTGTAGCCAA** |
| Mus-U6-Forward | **CTCGCTTCGGCAGCACA** |
| Mus-U6-Reverse  Human-U6-Forward  Human-U6-Reverse | **AACGCTTCACGAATTTGCGT**  **CTCGCTTCGGCAGCACAT**  **TTTGCGTGTCATCCTTGCG** |
| Mus-Lnc-PAR-Forward | **ACCAAGGTTGGTGGAGGAAG** |
| Mus-Lnc-PAR-Reverse | **GGTCTCTAGTAGGGCCGGT** |
| Mus-miR-141-5P-Forward | **AACACTGTCTGGTAAAGATGG** |
| Mus-miR-141-5P-Reverse  Human-miR-141-5P-Forward  Human-miR-141-5P-Reverse | **CTGTCTGGTAAAGATGGCCC**  **CTTCCAGTACAGTGTTGG**  **GAACATGTCTGCGTATCTC** |
| Mus-pre-miR-141-Forward | **GGGTCCATCTTCCAGTGCAGT** |
| Mus-pre-miR-141-Reverse | **CTGTCTGGTAAAGATGGCCC** |

**Table S4. Primary antibodies for WB, IHC and IF**

| **Antibody** | **Concentration**  **for WB** | **Concentration**  **for IHC** | **Concentration**  **for IF** | **Specificity** | **Company** |
| --- | --- | --- | --- | --- | --- |
| **Fibronectin** | **1:1000** | **1:100** |  | **Rabbit polyclonal** | **Proteintech** |
| **RB1CC1** | **1:1000** | **1:200** |  | **Rabbit polyclonal** | **Proteintech** |
| **Collagen III** | **1:1000** | **1:200** |  | **Rabbit polyclonal** | **Proteintech** |
| **Collagen I** | **1:1000** | **1:200** |  | **Rabbit polyclonal** | **Proteintech** |
| **α-SMA** | **1:2000** | **1:200** | **1:200** | **Rabbit mAb** | **Cell**  **Signaling Technology** |
| **LC3B** | **1:2000** | **1:200** |  | **Rabbit polyclonal** | **Proteintech** |
| **P62** | **1:2000** |  |  | **Rabbit polyclonal** | **Proteintech** |
| **GAPDH** | **1:2000** |  |  | **Mouse monoclonal** | **Kangcheng** |

**Figure Legends**

**Fig. S1** (A) The efficiency of sh-Lnc-PFAR in PSCs cell lines. (B-C) The expressions of α-SMA, Collagen I, Collagen III, Fibronectin were tested in PSCs in Lnc-PFAR downregulated or upregulated PSCs. The relative expression represents the ratio of target to GAPDH. (D-E) The α-SMA expressions in 1% area of IF in different groups. The results are representative of three independent experiments (**p* < 0.05, ***p* < 0.01 and ns *p* > 0.05).

**Fig. S2** (A-B) The expressions of Lnc-PFAR were tested in primary PSCs treated with TGF-β combined with downregulated or upregulated Lnc-PFAR. (C-D) The α-SMA expressions were explored in primary PSCs of quiescent, TGF-β-treated, sh-Lnc-PFAR, sh-Lnc-PFAR plus TGF-β, Lnc-PFAR-up-regulated and Lnc-PFAR plus TGF-β groups via immunofluorescence staining (bars, 500μm). (E-F) The α-SMA expressions in 1% area in different groups. The results are representative of three independent experiments (**p* < 0.05, ***p* < 0.01 and ns *p* > 0.05).

**Fig. S3** (A-B) The efficiency of miR-141-5P mimic and inhibitor. The results are representative of three independent experiments (**p* < 0.05, ***p* < 0.01 and ns *p* > 0.05).

**Fig. S4** (A-B) The quantifications of RFP and mRFP dots were determined by fluorescent puncta.

**Fig. S5** (A) The schematic view of animal study. (B-G) The expression levels of Lnc-PFAR, pre-miR-141 and miR-141-5P were explored in plasma of negative control, CP, CP plus sh-Lnc-PFAR injection, and CP plus Lnc-PFAR injection groups.

**Fig. S6** (A) The correlations of plasma Lnc-PFAR, pre-miR-141 and miR-141-5P levels in sh-Lnc-PFAR lentivirus injection group. (B) The correlations of plasma Lnc-PFAR, pre-miR-141 and miR-141-5P levels in Lnc-PFAR injection group. (C) The correlations of tissue Lnc-PFAR, pre-miR-141 and miR-141-5P levels in sh-Lnc-PFAR injection group. (D) The correlations of tissue Lnc-PFAR, pre-miR-141 and miR-141-5P levels in Lnc-PFAR injection group.

**Fig. S7** (A-B) The expressions of α-SMA, Collagen I, Collagen III, Fibronectin, RB1CC1, P62 and LC3Ⅱ/Ⅰ were tested in negative control, CP, sh-Lnc-PFAR, and CP plus sh-Lnc-PFAR, Lnc-PFAR and CP plus Lnc-PFAR groups. The relative expression represents the ratio of target to GAPDH. The results are representative of three independent experiments (**p* <0.05, ***p* < 0.01 and ns *p* > 0.05). (C, E) The expression level of α-SMA and the correlation between α-SMA and Lnc-PFAR in pancreatic tissue. (D, F) The expression level of α-SMA and the correlation between α-SMA and Lnc-PFAR in plasma.

**Fig. S8** (A-C) The expression levels of Lnc-PFAR, pre-miR-141 and miR-141-5P were explored in pancreatic tissues of negative control and caerulein injection CP model groups. (D) The H&E and Masson staining were conducted to indicate CP and pancreatic fibrosis in mice models of negative control and caerulein injection CP model groups (n = 5) (original magnification, 20×, bars, 50μm).

**Fig. S9** (A-B) The correlation of tissue Lnc-PFAR, pre-miR-141 and miR-141-5P levels in different groups. (C) The expression levels of Lnc-PFAR were explored in pancreatic tissues of chronic pancreatitis and pancreatic cancer groups.

**Fig. S10** (A-B) The proliferation rate of PSCs was analyzed by CCK-8 assay of in different groups (NC, TGF-β, sh-Lnc-PFAR, sh-Lnc-PFAR plus TGF-β, Lnc-PFAR and Lnc-PFAR plus TGF-β). (C-F) The migration ability of activated PSCs was demonstrated by Transwell assay (bars, 50 μm).

**Fig. S11** (A) FISH assay demonstrated that miR-141 and Lnc-PFAR were mutually exclusive in cytoplasm in quiescent and activated primary PSCs. (B) FISH assay indicated the co-localization between Lnc-PFAR and pre-miR-141 in quiescent and activated primary PSCs (bars, 500μm).

**Supplementary methods**

**PSCs isolation**

Primary PSCs were isolated from murine pancreas with 0.03% collagenase P (Roche, Germany) digestion and Nycodenz density gradient centrifugation. Primary PSCs were cultured in Dulbecco’s modified Eagle's medium (Gibco, Gaithersburg, USA), supplemented with 10% fetal bovine serum (Gibco) at 37°C with 5% CO_2_. The medium was changed and the contaminating cells were removed at 24 h after seeding. Primary PSCs were used before the first passage.

Migration assay

Migration was performed as described previously^1^. Culture medium containing 10% FBS was added to lower chambers and 5-10 × 10^4^ cells in 200μl of FBS-free medium were seeded into upper chambers. After incubation for 24 h, non-migrated cells were removed from the upper surface of the filter with a cotton swab. Invasive cells on the bottom surface of the membrane were fixed in methanol and then stained with crystal violet. The number of cells in five randomly selected fields was counted.

CCK-8 assay

Cell proliferation was examined using Cell Counting Kit-8 (CCK-8, Dojindo Laboratories, Kumamoto, Japan) according to the manufacturer’s instructions. PSCs (5×10^3^ cells per well) were seeded into a 96-well plate. Absorbance was measured daily for 3 consecutive days at 450nm (ELx808, BioTek, USA).

**Transfection**

PSCs were equally distributed into a 6-well plate with 2-3×10^5^ cells per well. The 50 nM negative control, 50 nM siRNA of Lnc-PFAR, 50nM miR-141-5P mimics or 100nM miR-141-5p inhibitor (Ribobio, Guangzhou, China) were transfected into PSCs using Lipofectamine 2000 (Invitrogen, Carlsbad, CA, USA) according to the manufacture’s protocol when cells were 30-50% confluent. After 24 to 48h, the transfected cells were harvested for subsequent experiments. The qRT-PCR analysis was performed to analyze transfection efficacy.
**Reference:**

1. Li L, Chen H, Gao Y, Wang YW, Zhang GQ, Pan SH, et al. Long Noncoding RNA MALAT1 Promotes Aggressive Pancreatic Cancer Proliferation and Metastasis via the Stimulation of Autophagy. *Mol Cancer Ther* **15**, 2232-2243 (2016).

**Ethical support for this experiment**


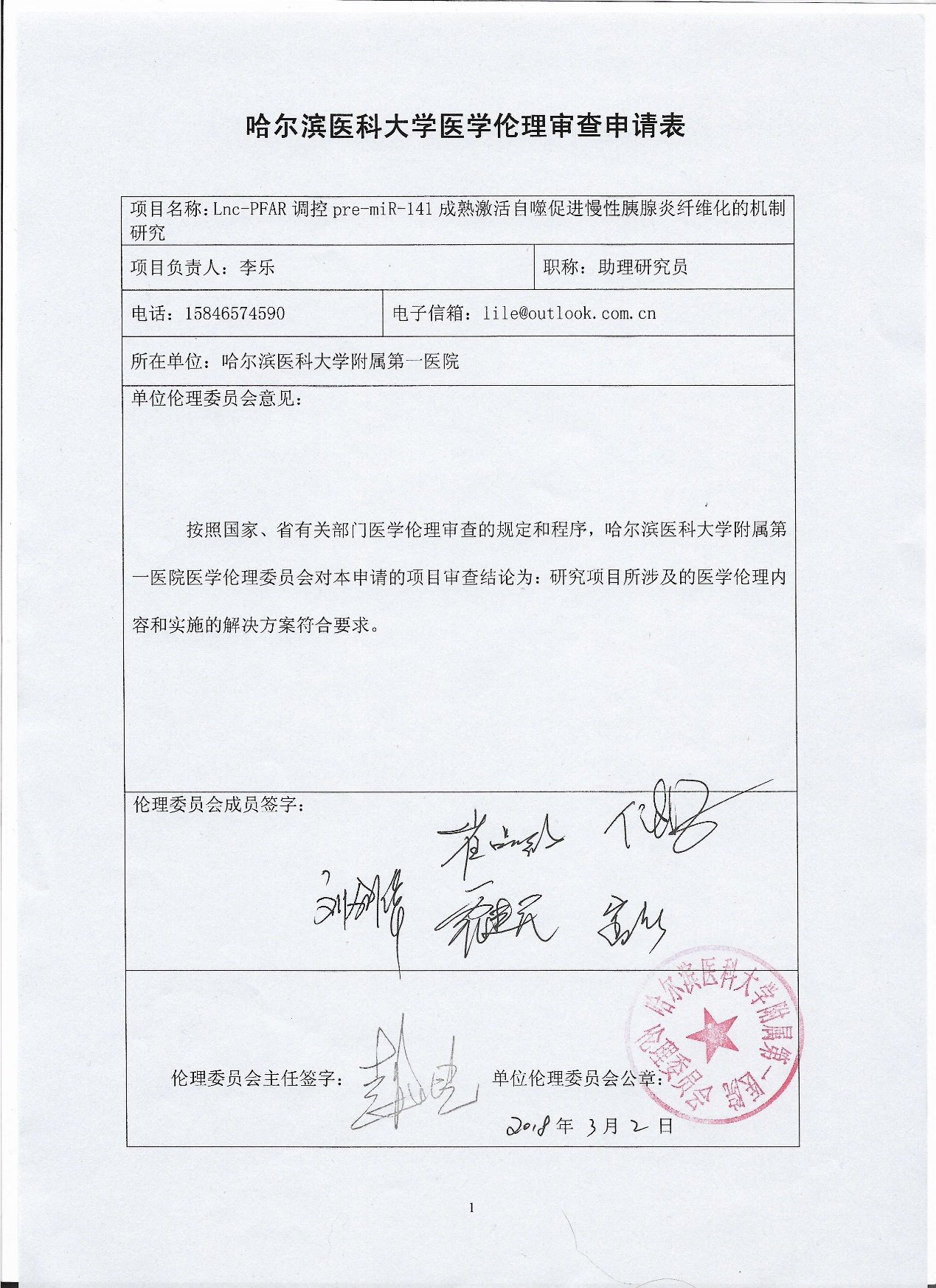

Supplement: Supplementary file 1 — supplemental information [file 41419_2021_4236_MOESM1_ESM.docx]
