# Supplementary figures and images for: Lnc-PFAR facilitates autophagy and exacerbates pancreatic fibrosis by reducing pre-miR-141 maturation in chronic pancreatitis
Source: Cell Death Dis. 2021 Oct 25;12(11):996. doi: 10.1038/s41419-021-04236-z (PMC8547218; doi:10.1038/s41419-021-04236-z)

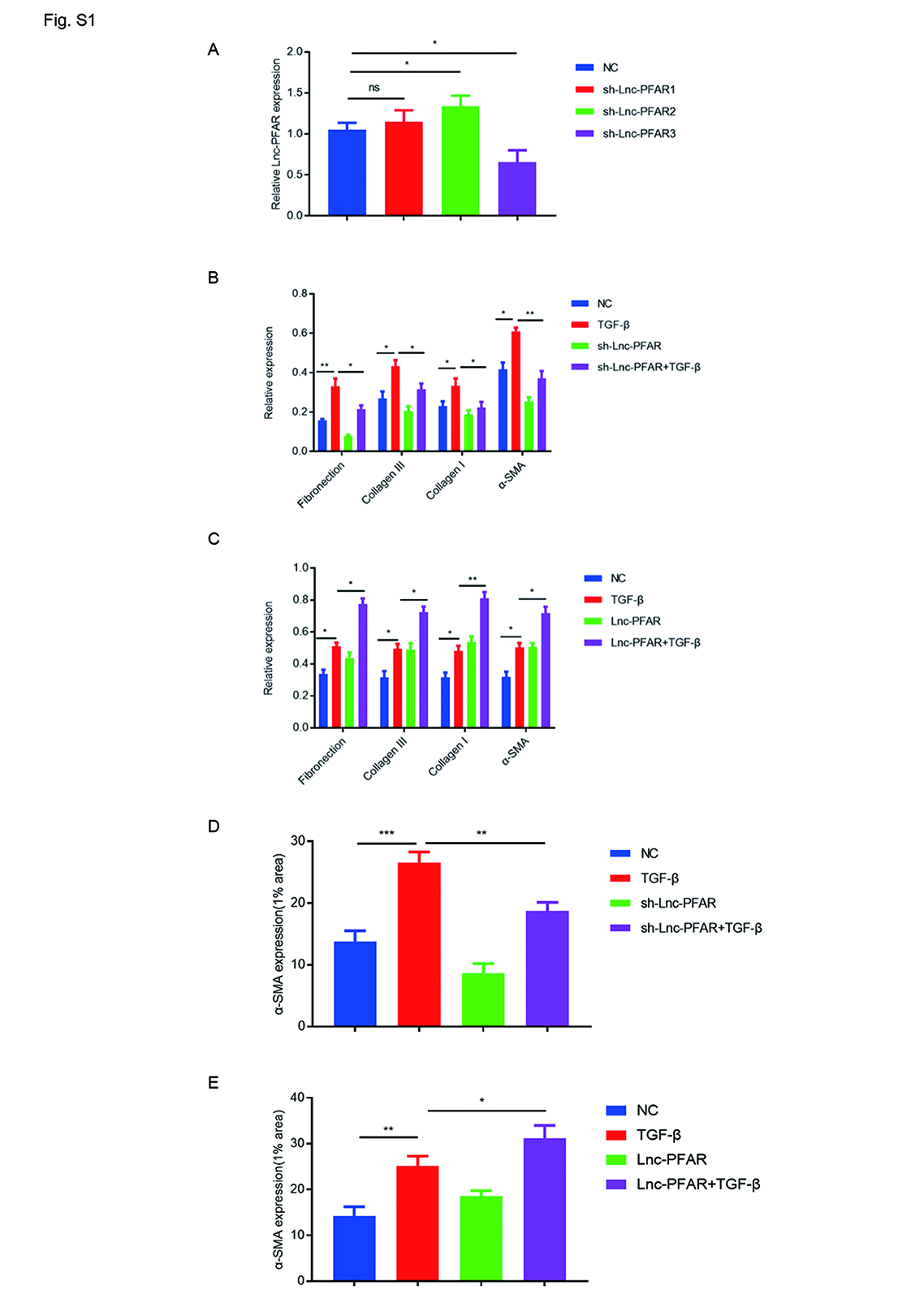

Supplement: Supplementary file 2 — Figure S1 [file 41419_2021_4236_MOESM2_ESM.tif]

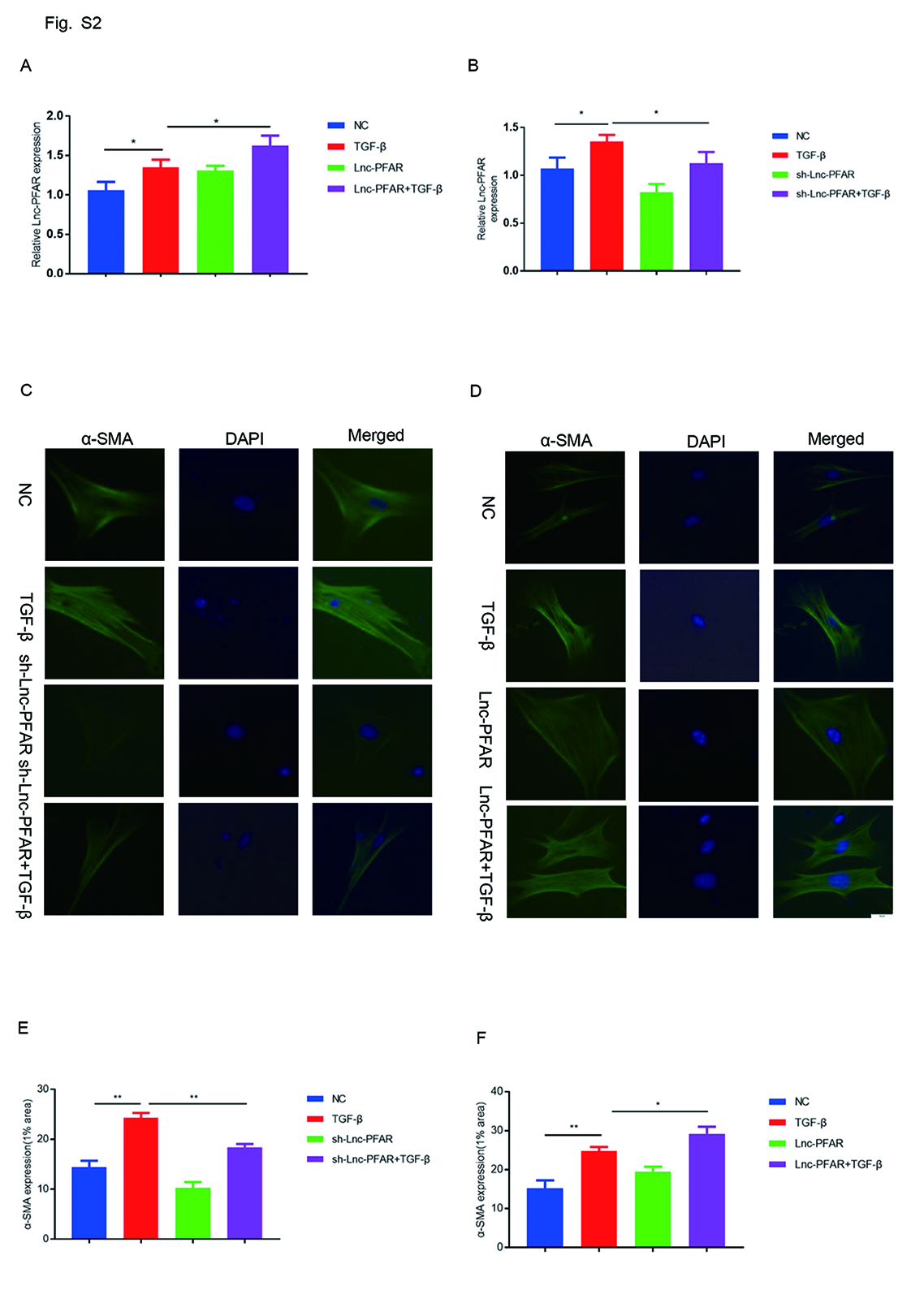

Supplement: Supplementary file 3 — Figure S2 [file 41419_2021_4236_MOESM3_ESM.tif]

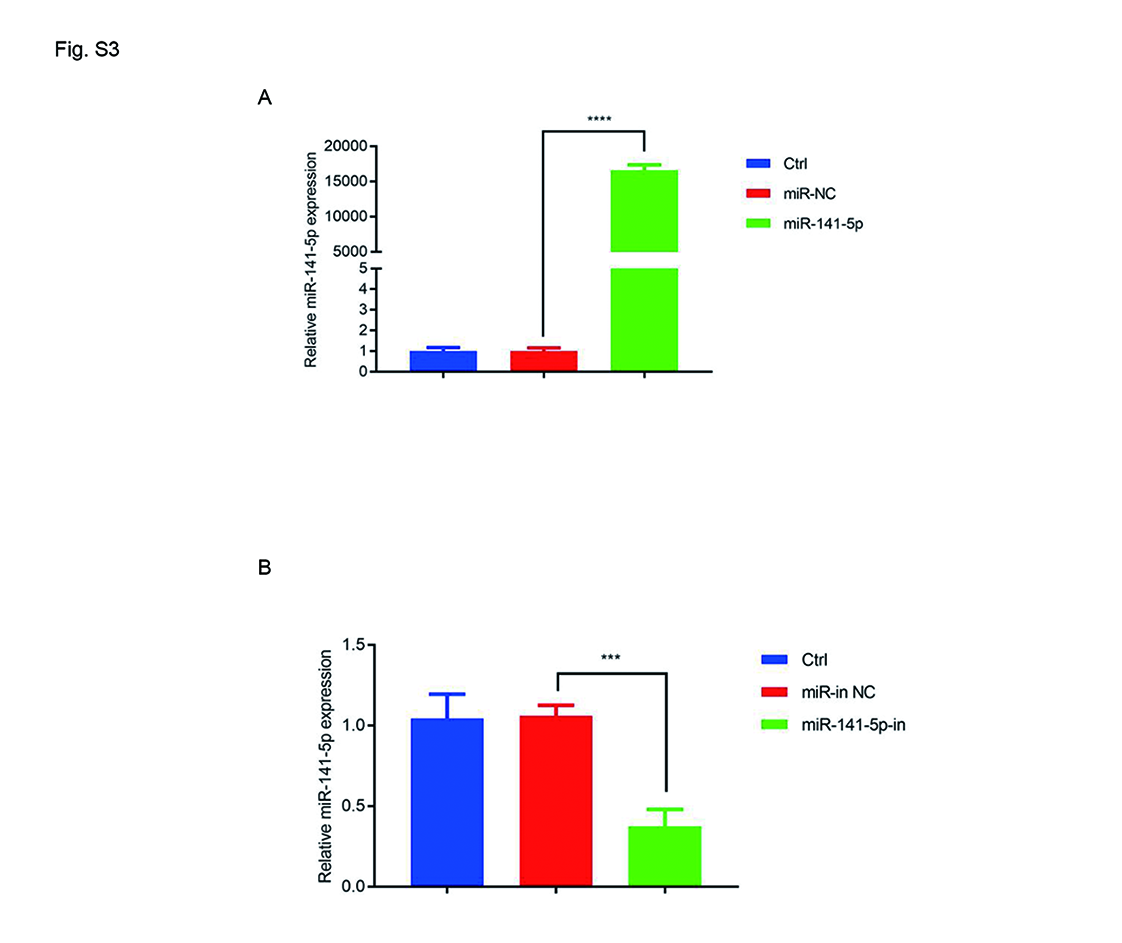

Supplement: Supplementary file 4 — Figure S3 [file 41419_2021_4236_MOESM4_ESM.tif]

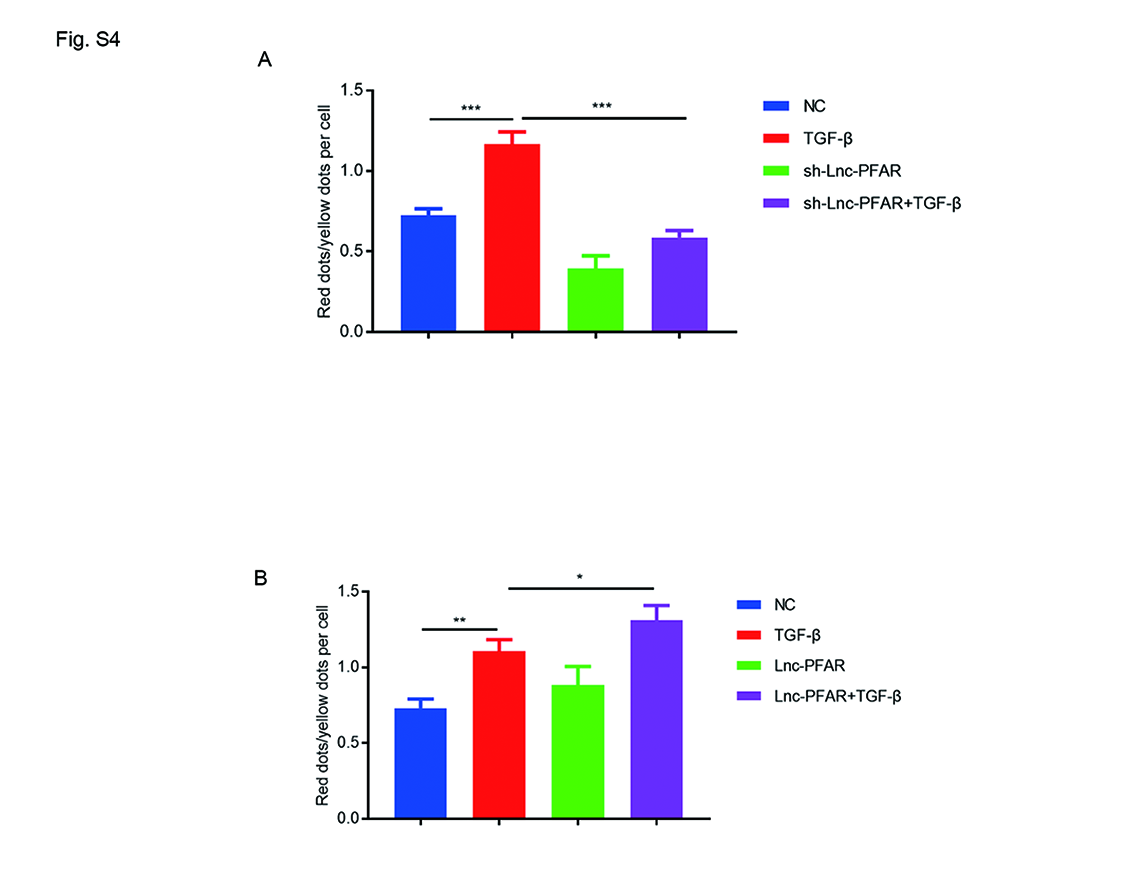

Supplement: Supplementary file 5 — Figure S4 [file 41419_2021_4236_MOESM5_ESM.tif]

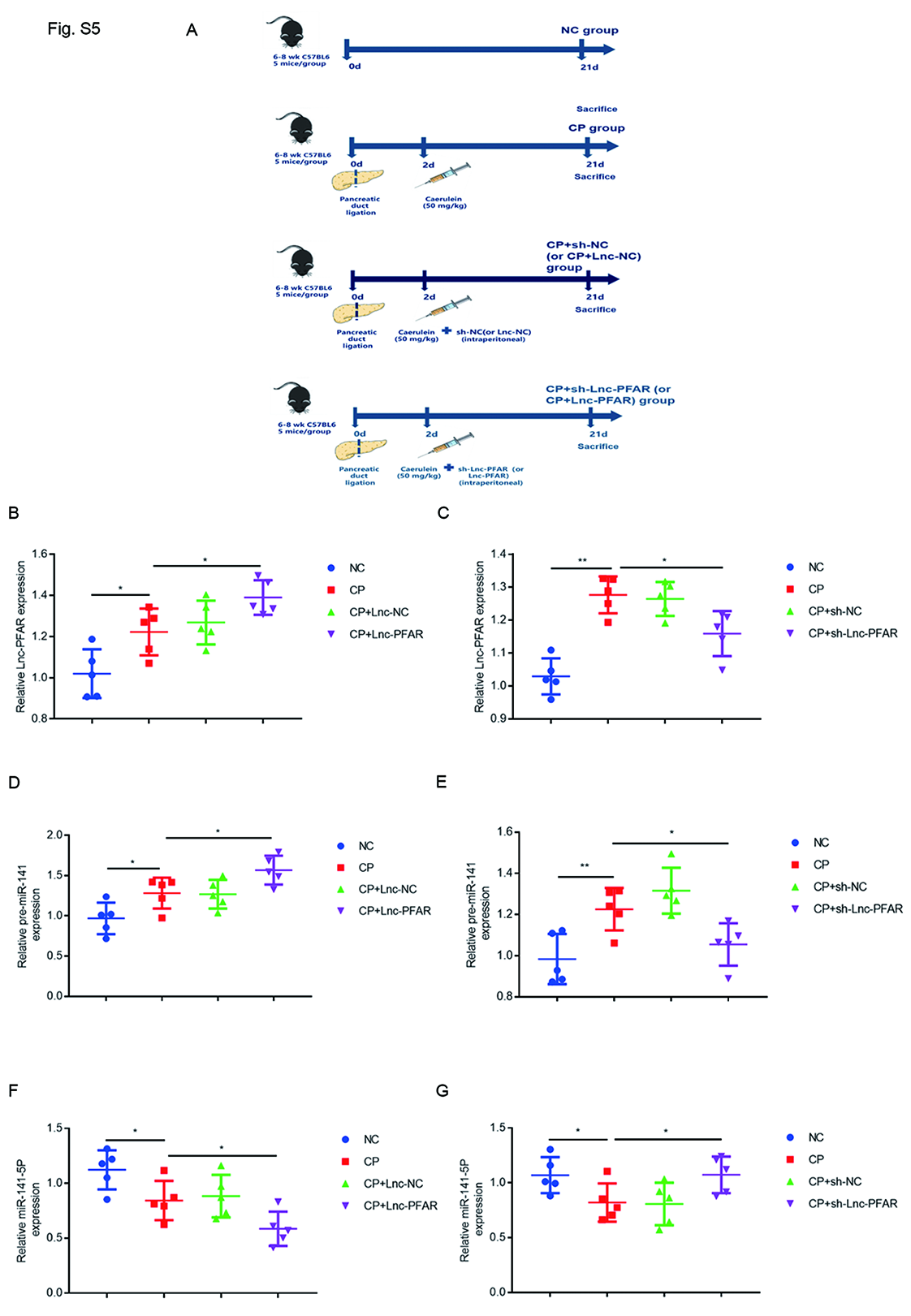

Supplement: Supplementary file 6 — Figure S5 [file 41419_2021_4236_MOESM6_ESM.tif]

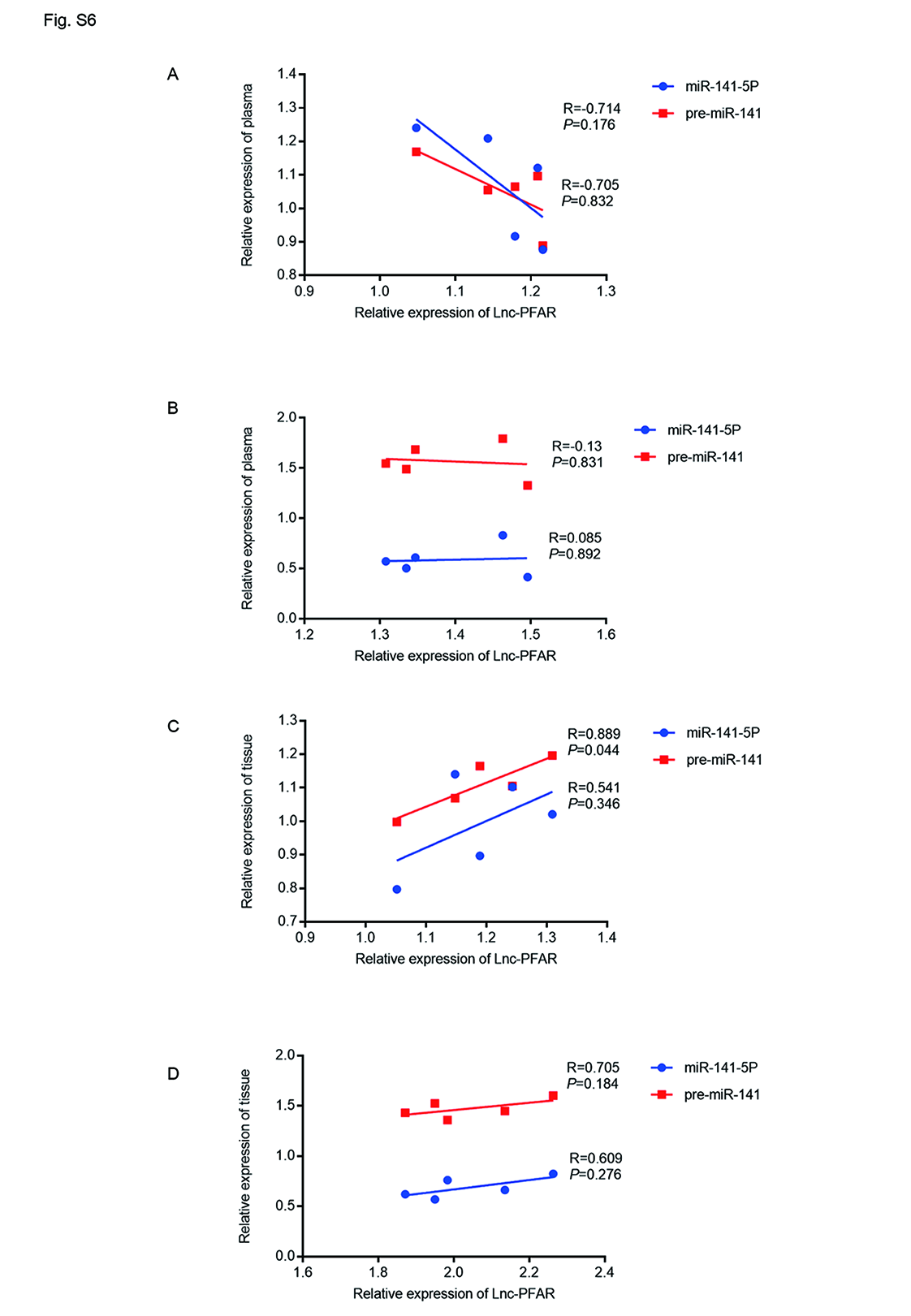

Supplement: Supplementary file 7 — Figure S6 [file 41419_2021_4236_MOESM7_ESM.tif]

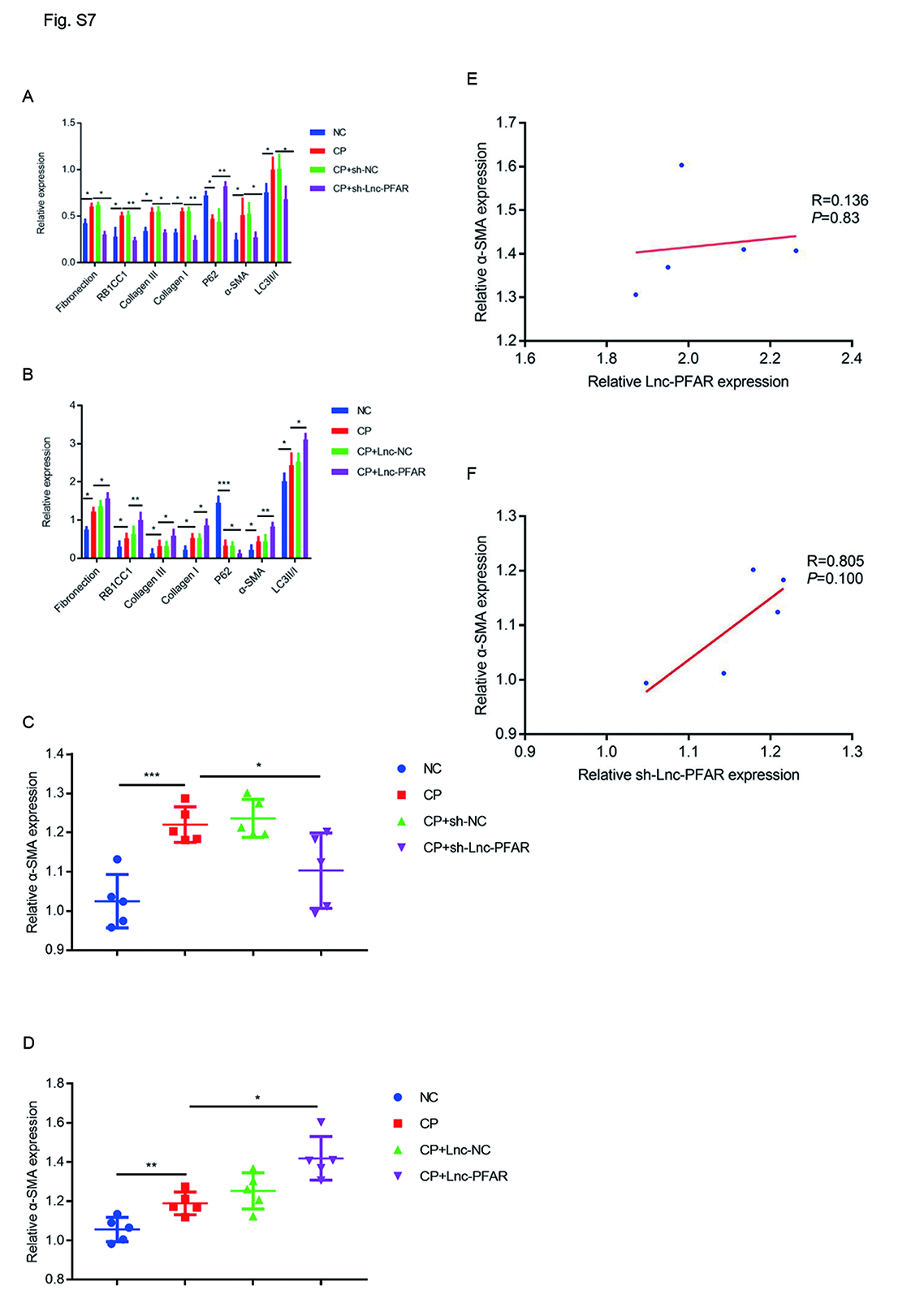

Supplement: Supplementary file 8 — Figure S7 [file 41419_2021_4236_MOESM8_ESM.tif]

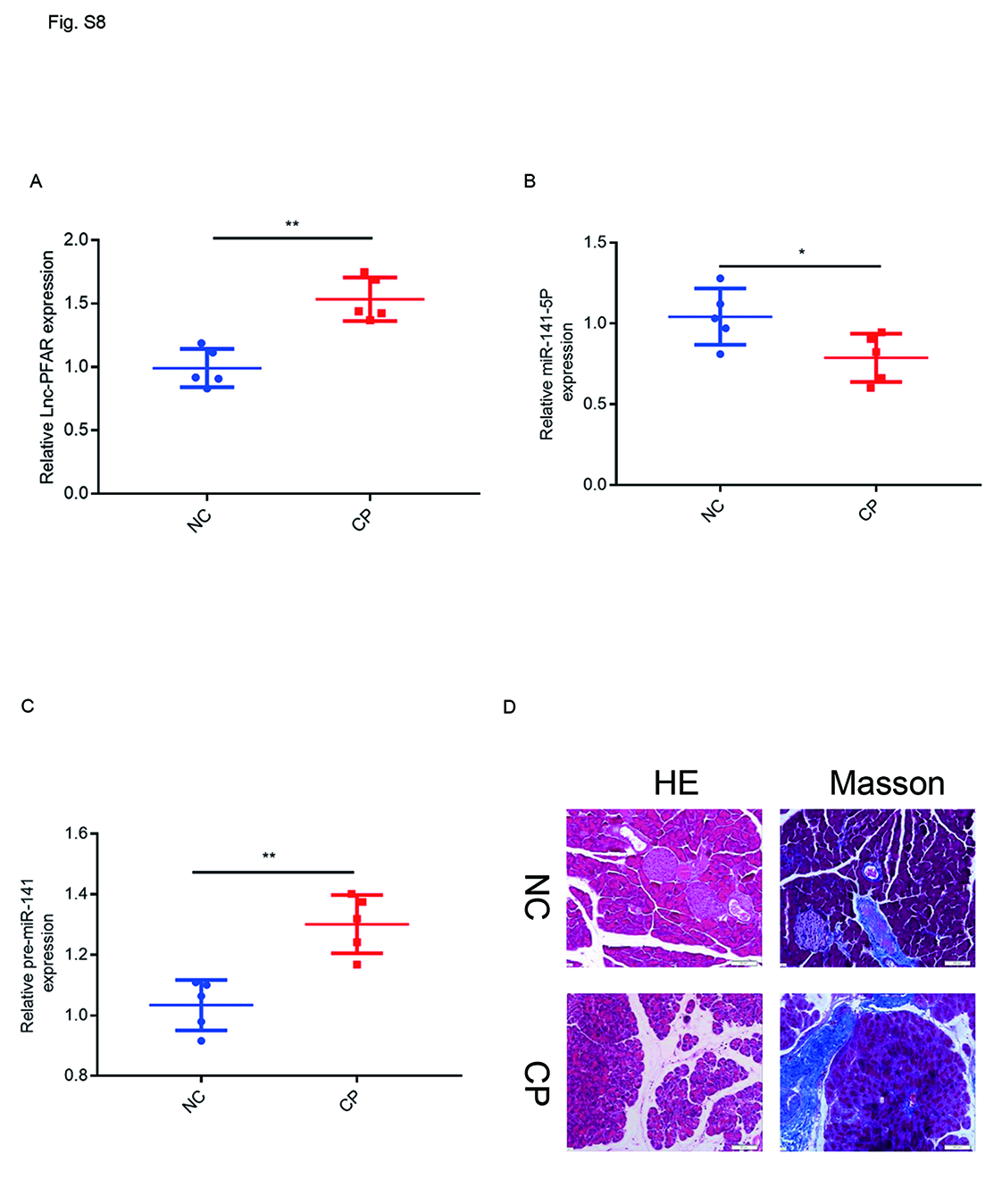

Supplement: Supplementary file 9 — Figure S8 [file 41419_2021_4236_MOESM9_ESM.tif]

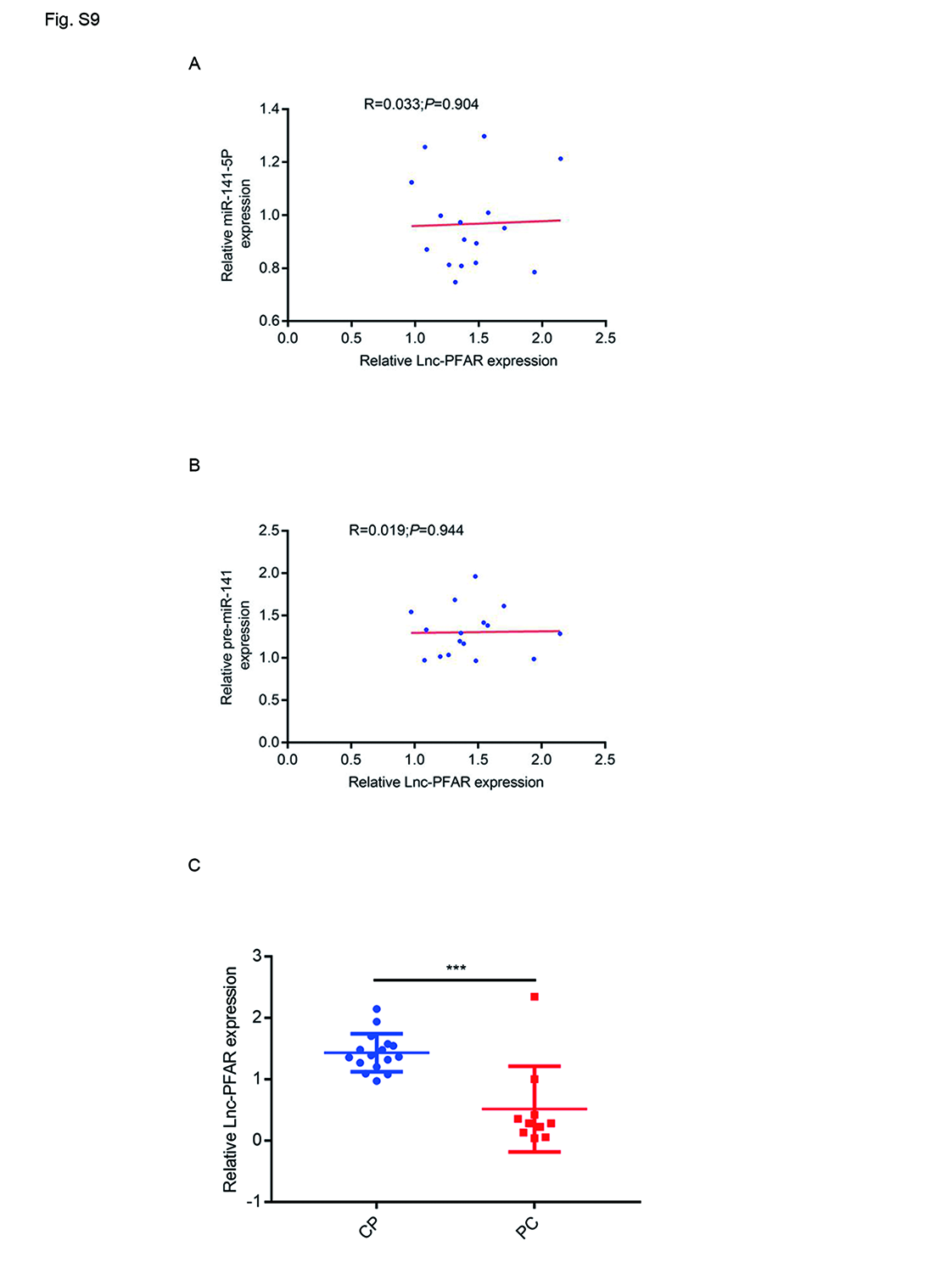

Supplement: Supplementary file 10 — Figure S9 [file 41419_2021_4236_MOESM10_ESM.tif]

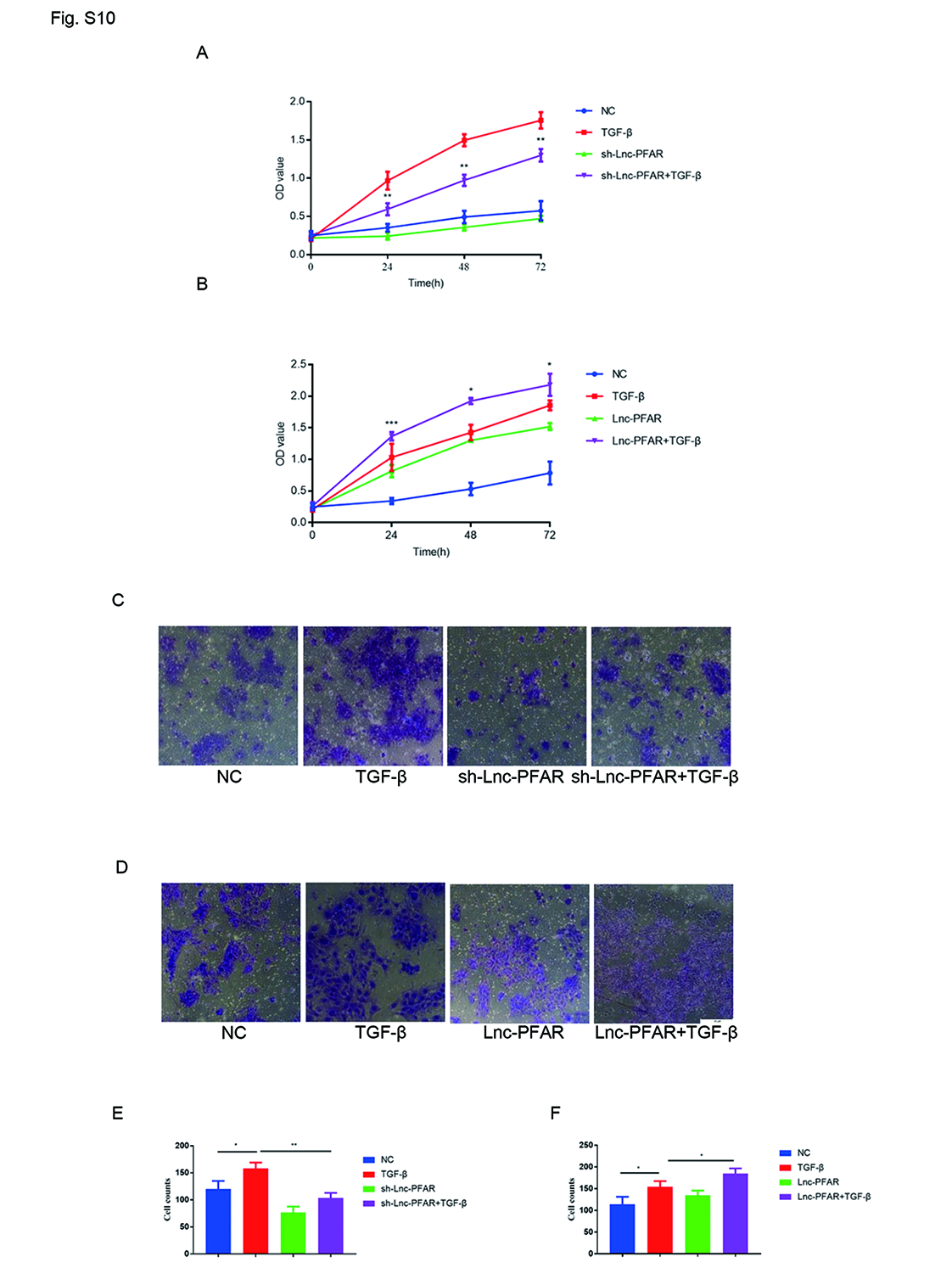

Supplement: Supplementary file 11 — Figure S10 [file 41419_2021_4236_MOESM11_ESM.tif]

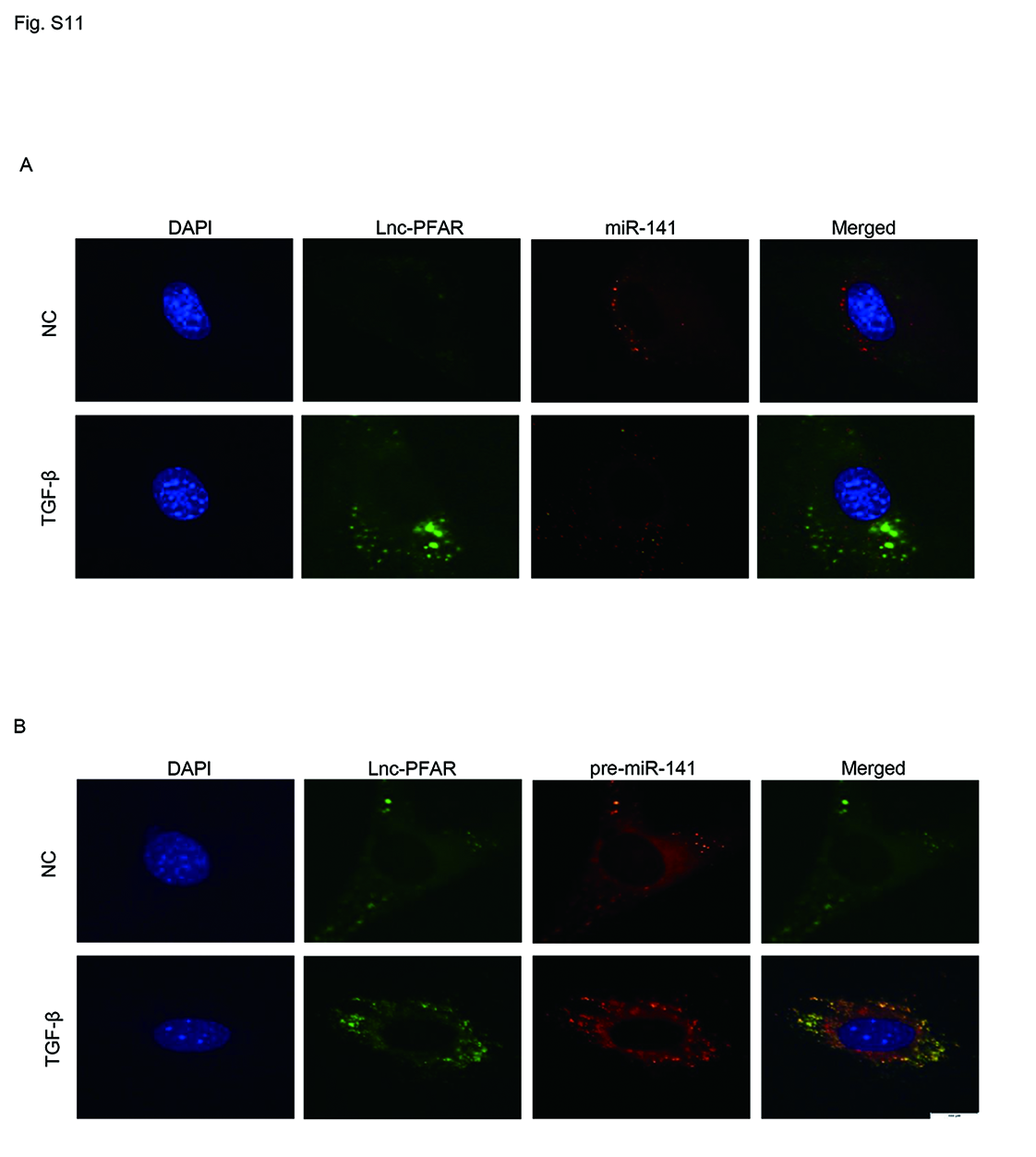

Supplement: Supplementary file 12 — Figure S11 [file 41419_2021_4236_MOESM12_ESM.tif]
